# Enabling High‐Performance Electrode Materials for Potassium‐Ion Batteries: Ionic Transport, Size and Electro–Chemo–Mechanical Effects

**DOI:** 10.1002/advs.202509543

**Published:** 2025-08-13

**Authors:** Changbao Zhu, Yanpeng Fu, Yong Yang

**Affiliations:** ^1^ Guangdong Provincial Key Laboratory of Sensing Physics and System Integration Applications School of Physics and Optoelectronic Engineering Guangdong University of Technology Guangzhou 510006 China; ^2^ Institute of Batteries School of Materials and Energy Guangdong University of Technology Guangzhou 510006 China; ^3^ State Key Laboratory for Physical Chemistry of Solid Surfaces Collaborative Innovation Center of Chemistry for Energy Materials and Department of Chemistry College of Chemistry and Chemical Engineering Xiamen University Xiamen 361005 China

**Keywords:** electro–chemo–mechanical behavior, electrode materials, potassium‐ion batteries, size effects, transport properties

## Abstract

Potassium‐ion batteries, which possess unique advantages such as lower K^+^/K redox potential compared to sodium and superior interfacial charge transfer dynamics, demonstrate considerable viability for grid‐level energy storage deployment. However, the development of potassium electrodes remains constrained by sluggish solid‐state diffusion of K^+^ within electrodes and progressive structural failure by the large volume variation during (de)intercalation, which requires a thorough understanding ionic transport, size effects, and electro–chemo–mechanical properties of electrodes, to achieve rational design and controlled synthesis. This review initiated with a comprehensive evaluation of potassium‐based batteries from five aspects: energy density, power density, cycle life, safety, and cost. Afterward, a systematical examination for key aspects of potassium electrodes from a unique perspective is provided, starting with the fundamental scientific issues of transport properties (key features, anomalous cases, regulation, measurement and prediction) and size effects (kinetics, thermodynamics, potassium storage and transport mechanisms), while further discussing the specific electro–chemo–mechanical properties (composition–structure regulation, nanostructure and interface engineering). Additionally, this review highlights the construction of high‐entropy electrodes and the pivotal role of machine learning in developing potassium electrodes. This review aims to provide critical guidance for future basic research and industrial applications of potassium electrode materials.

## Graphical abstract

Potassium‐ion battery is a promising candidate for large‐scale energy storage applications. In this review, transport properties, size effects, and electro–chemo–mechanical properties of electrodes are systematically summarized in order to achieve the rational design and controlled synthesis of potassium electrodes.

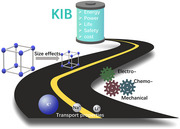

## 1 Introduction

Li‐ion battery systems have achieved significant commercial success.^[^ ^1^ ^]^ However, the limited geographical distribution and high price of lithium reserves have constrained further advancements in related industries, especially for grid‐level energy storage.^[^ ^2^ ^]^ Price‐competitive Na‐ or K‐ion batteries demonstrate substantial application potential.^[^ ^3^, ^4^, ^5^ ^]^ Among these, Na‐ion batteries have made great progress toward industrialization, whereas K‐ion batteries remain in the early stages of development. Despite this, K‐ion batteries possess unique advantages, including a lower K^+^/K redox potential and superior interfacial charge transfer dynamics. Consequently, K‐ion batteries and rechargeable potassium‐based systems have emerged as frontiers of investigation globally.^[^ ^6^, ^7^ ^]^

**Figure 1:**
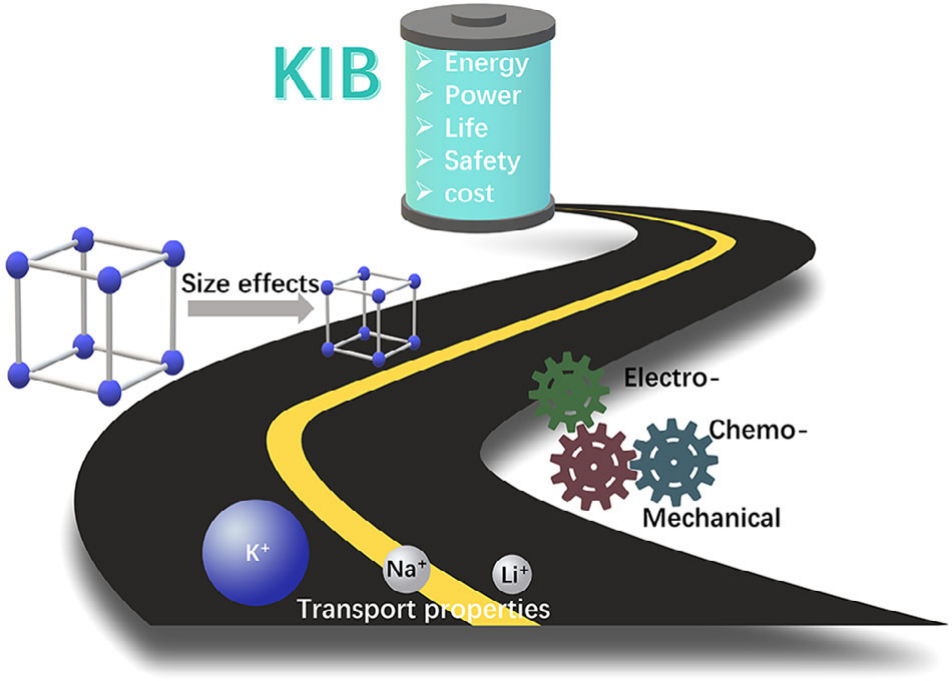
Schematic diagram of the main contents and key points reviewed in this paper.

However, given the higher molecular weight and larger ionic radius of K^+^, it exhibits distinct advantages and challenges in potassium systems compared to lithium and sodium systems. This review aims to systematically evaluate the advantages and limitations of K‐ion batteries relative to Li (Na)‐ion batteries, thereby indicating potential development directions. As to potassium electrode materials, compared with lithium and sodium counterparts, their larger ion size leads to poor transport characteristics and significant volume changes during (de)intercalation, which are critical issues that need to be addressed. The size effect is an effective strategy for resolving the aforementioned problems. This review aims to elucidate the key scientific issues in designing high‐performance potassium electrode materials by focusing on ionic transport, size effects, and electro–chemo–mechanical properties. The deep understanding of transport characteristics and size effects establishes the basis, while precise elucidation of the electro–chemo–mechanical properties holds special significance for large‐sized potassium‐ion intercalation. From the perspective of ionic transport and size effects, this review highlights their importance in comprehending, modifying, and developing electrode materials while further discussing the specific electro–chemo–mechanical behaviors of potassium electrodes (**Figure** **1**). Regarding transport characteristics, this review summarizes the key features, basic requirements, anomalous cases, intrinsic transport regulation, and methods for measuring and predicting transport characteristics in potassium electrodes, with particular emphasis on the significant role of high‐entropy electrodes and machine learning. In terms of size effects, the influence of size on kinetics, thermodynamics (including equilibrium potential, reversibility, intrinsic stress characteristics), as well as potassium storage and transport mechanisms (e.g., interface effect, hierarchically porous structures, amorphous structure, phase transition mechanism) is thoroughly discussed. In addition, the electro–chemo–mechanical coupling effects of potassium electrodes can be improved through strategies such as composition–structure regulation (e.g., doping, high‐entropy electrode design), nanostructure engineering (e.g., nanoscale design, porous structures, amorphous phases, phase transition mechanism regulation), and interface engineering (e.g., charging electrolyte interface (CEI)/solid electrolyte interface (SEI) composition‐mechanical strength optimization).

## 2 Comprehensive Evaluation of Potassium‐Based Batteries

We will make a thorough evaluation of potassium‐ion batteries and other potassium‐based systems, assessing them from five critical aspects: energy density, power density, cycle life, safety, and cost. Particular emphasis will be placed on comparing these properties with those of corresponding lithium and sodium systems. Detailed comparative data for lithium, sodium, and potassium, including their physical and chemical aspects, are presented in **Table** **1**.^[^ ^4^, ^8^, ^9^, ^10^, ^11^, ^12^, ^13^, ^14^ ^]^

**Table 1:** Comparison of the chemical and physical properties of Li, Na and K, as well as corresponding energy storage systems.

Physical and chemical properties	Lithium	Sodium	Potassium
Relative atomic mass	6.941	22.989	39.098
Ionic radius [Å]	0.76	1.02	1.38
Melting point [°C]	180.5	97.7	63.4
Theoretical capacity [mAh g^−1^]	3827	1165	685
Abundance in earth crust [wt.%]	0.0017	2.3	1.5
Concentration in seawater [mg L^−1^]	0.1	10 556	380
Density [g cm^−3^]	0.535	0.968	0.856
Cost of carbonate [$ ton^−1^]	6500	200	1000
Distribution	70% in South America	Everywhere	Everywhere
Alloying with Al	Yes	No	No
Shear modulus [GPa]	4.1	3.3	1.3
E^0^ vs SHE [V]	−3.04	−2.71	−2.93
E^0^ vs SHE in PC [V]	−2.79	−2.56	−2.88
Stokes radius [Å] in PC	4.8	4.6	3.6
Desolvation energy in PC [KJ mol^−1^]	215.8	158.2	119.2
Intercalation in graphite	LiC_6_	NaC_16_, not stable	KC_8_
Ionic conductivity of 1 m Li/Na/KFSI in EC/DEC [mS cm^−1^]	1	9.7	10.7
Coordination numbers in inorganic solid‐state electrolytes	3 or 4 in sulfides, 4 or 6 in oxides	6, 7 or 8	8, 9 or 12
Diffusion path in inorganic solid‐state electrolytes	Triangle	Quadrangle	Hexagon

From the perspective of energy density, we conduct a comparative analysis based on two aspects: capacity and voltage. Due to the gradual increase in atomic mass from lithium to sodium and then to potassium, the alkali metal series’ theoretical capacities are 3827, 1165, and 685 mAh g^−1^, respectively. For potassium‐based batteries using an alkali metal anode, the capacity decreases significantly from lithium to sodium to potassium. However, in practical alkali metal ion batteries, metallic anodes have not been used. Instead, potassium ions are primarily stored in cathode and anode materials, operating as a rocking chair battery. Consequently, although the actual K‐ion electrode capacity is lower compared to those of Li or Na ion electrodes, the difference is not substantial.^[^ ^13^ ^]^ Moreover, energy density is less critical for grid‐level energy storage, which makes price‐competitive sodium and potassium systems promising candidates. Within the alkali metal electrochemical series, potassium exhibits a redox potential of −2.93 V (vs SHE)—a modest 110 mV positive shift relative to lithium (−3.04 V), yet remains 220 mV more reducing than sodium (−2.71 V) under standard conditions. This allows potassium‐ion batteries to potentially achieve higher voltages than sodium‐ion batteries, thereby enhancing their energy density. Additionally, metallic potassium even demonstrates a reduction in equilibrium potential relative to lithium counterparts in propylene carbonate (PC) electrolyte (Table 1),^[^ ^10^ ^]^ further highlighting the significant potential for developing high‐voltage K‐ion batteries. Therefore, K‐ion batteries have the potential to match or even exceed Na‐based systems in energy density.

As to power density (rate performance), the Li^+^, Na^+^, K^+^ transport properties in electrode materials, electrolytes, and interfaces are critical determinants of their rate performance. For electrode materials, as the ion radius increases from Li^+^ (0.76 Å) to Na^+^ (1.02 Å) to K^+^ (1.38 Å), the ion transport properties progressively deteriorate within the electrodes, leading to a decreased diffusion coefficient. For instance, the K^+^ diffusion coefficient is reduced than that of Na^+^ in hard carbon.^[^ ^15^ ^]^ Therefore, enhancing the transport characteristics of potassium ions in potassium‐based electrode materials is crucial. On the other hand, potassium ions exhibit advantages over lithium and sodium ions in terms of electrolyte transport and interface charge transfer. The larger size of potassium ions results in lower charge density, reduced Lewis acidity, weaker interactions with solvent molecules, and lower desolvation energy, which facilitates faster interfacial charge transfer kinetics. Additionally, in propylene carbonate (PC) solvents, the Stokes radius of K^+^ (3.6 Å) is significantly smaller than that of lithium ions (4.4 Å) and sodium ions (4.6 Å), enabling rapid transport and higher ionic conductivity in organic electrolytes (Table 1).^[^ ^8^, ^11^, ^13^ ^]^ Consequently, K‐ion batteries may exhibit superior rate performance versus Li‐ion and Na‐ion batteries. The key point lies in leveraging the advantages of potassium ion transport in the electrolyte and interface while improving transport properties in the electrode material.

From the perspective of cycle life, K‐ion batteries show inferior performance versus Na‐ion batteries owing to the higher reduction tendency and activity of potassium. In ester‐based electrolytes, this leads to the reduction of the electrolyte, triggering various side reactions that result in poor cycling stability, low Coulombic efficiency, and potentially causing electrolyte dry‐out and battery failure.^[^ ^16^, ^17^, ^18^ ^]^ For the potassium electrode, the repeated (de)intercalation of large potassium ions induces significant volume variations in the electrode material,^[^ ^19^ ^]^ leading to electrode structural damage or even electrode pulverization, which sharply degrades cycle performance. For instance, for graphite, the volume variation due to K^+^ intercalation is six times greater than that caused by lithium ion intercalation.^[^ ^18^ ^]^ For conversion/alloying anode materials such as Sn_4_P_3_, the volume change after potassium storage can be as high as 680%.^[^ ^20^, ^21^ ^]^ The volume expansion and structural stress not only decrease the cycle life of the electrode material but also lead to the active material detachment from current collectors, as well as the degradation caused by electrolyte decomposition. Understanding the electro–chemo–mechanical properties of potassium electrodes is of great significance in the development of potassium electrodes. Introducing size effects, such as reducing the material sizes and constructing nanostructures with diverse morphologies, can effectively mitigate mechanical stress. This approach represents an effective strategy to enhance cycling performance, which will be particularly significant for K‐ion batteries versus Li‐ion and Na‐ion batteries.

From a safety perspective, potassium exhibits a significantly lower melting point compared to lithium and sodium, coupled with higher reactivity, which renders it more susceptible to side reactions and thermal runaway events, thereby posing greater safety risks. It indicates that while the potassium–graphite system generates less heat than its lithium–graphite counterpart, the onset temperature for thermal runaway is lower, necessitating vigilant attention to safety concerns.^[^ ^22^ ^]^ Moreover, due to potassium's low melting point, potassium dendrites can self‐heal via Joule heating more readily than lithium and sodium metal anodes.^[^ ^23^, ^24^ ^]^ Additionally, from a dendrite formation standpoint, as shown in Table 1, the shear modulus of K (1.3 GPa) is considerably lower than that of Li (4.1 GPa) and Na (3.3 GPa).^[^ ^4^ ^]^ Consequently, employing a mechanical pressure strategy can help maintain a smooth surface on the potassium metal anode, thereby enhancing the overall safety of potassium‐based batteries.

Finally, regarding cost considerations, K demonstrates notable crustal abundance at 1.5 mass%, slightly lower than Na (2.3%) yet three orders of magnitude greater than Li's scarcity (0.0017%). The abundance trend of Li, Na, and K in the ocean is similar to that observed in the Earth's crust (Table 1).^[^ ^6^ ^]^ K and Na are distributed uniformly all over the world, unlike lithium resources, which are predominantly concentrated in South America.^[^ ^14^ ^]^ This abundant availability renders K‐ion batteries similar to Na‐ion batteries, demonstrating substantial cost potential, suitable for energy storage applications. Furthermore, the absence of alloy formation between K/Na and Al enables aluminum foil to serve as an electrochemically stable anode current collector. This feature reduces manufacturing costs associated with inactive components, providing another critical advantage over lithium‐ion batteries.^[^ ^25^ ^]^ Additionally, given that lithium, sodium, and potassium show similar physicochemical properties, the production processes and equipment used for lithium ion batteries can serve as valuable references for Na‐ion and K‐ion battery technologies, facilitating industrialization efforts. Moreover, potassium ions exhibit an additional advantage over sodium ions by being able to intercalate effectively into graphite to form KC_8_, similar to lithium ions forming LiC_6_. In contrast, sodium ions cannot stably intercalate into graphite.^[^ ^26^ ^]^ Given that graphite is the most important lithium anode materials, its compatibility with potassium ions is highly beneficial for the development of commercial K‐ion batteries.

In addition, other potassium‐based systems also exhibit distinct advantages and challenges.^[^ ^27^ ^]^ For instance, potassium–sulfur^[^ ^28^, ^29^ ^]^ and potassium–oxygen batteries,^[^ ^30^, ^31^ ^]^ which utilize potassium metal as the anode, require focused research on the growth mechanisms and inhibition strategies of potassium dendrites. Compared to lithium and sodium, potassium's lower melting point, weaker shear strength, and higher reactivity necessitate particular attention. In aqueous potassium ion batteries, while the high ionic conductivity of potassium ions in aqueous solutions remains advantageous, efforts should be directed toward developing cathode and anode materials with suitable electrochemical windows^[^ ^32^ ^]^ to enhance energy density and facilitate potassium ion transport within electrode materials. For solid‐state potassium batteries, K^+^ (1.38 Å) with a large radius poses challenges for migration in solid electrolytes, limiting the available solid electrolyte structures.^[^ ^9^ ^]^ The coordination numbers and diffusion paths of K, Na, Li in their corresponding inorganic solid electrolytes are compared in Table 1.^[^ ^9^, ^33^, ^34^, ^35^, ^36^ ^]^ This limitation is more pronounced compared to Li‐ion batteries, where the larger sizes of Na^+^ and K^+^ restrict the development of inorganic oxide solid electrolytes. However, for potassium‐ion solid electrolytes, larger cations and expanded lattice structures can promote high ionic conductivities (3D superionic conductors).^[^ ^37^ ^]^ Additionally, incorporating larger anions or anion clusters can aid in designing solid electrolytes for sodium and potassium ions.^[^ ^38^ ^]^ Although single‐crystal K_2_Fe_4_O_7_ currently exhibits the highest room‐temperature potassium‐ion conductivity (with an ionic conductivity of 3.5 × 10^2^ S cm^−1^ and an activation energy of 0.08 eV),^[^ ^35^ ^]^ most of the known potassium‐ion conductors with distinctive room‐temperature conductivity are derivatives of lithium‐ or sodium‐ion conductors (such as K_3_SbS_4_, K‐β″‐Al_2_O_3_, K_3_OI).^[^ ^39^ ^]^ Potassium‐based solid electrolytes derived from lithium or sodium conductors typically require adjustments in lattice volume to accommodate the larger size of the K^+^. For instance, in the case of thiophosphate‐based solid electrolytes, the structural adaptation in K_3_SbS_4_, which originates from Na_3_PS_4_, is accomplished by substituting the smaller P^5+^ ions with the larger Sb^5+^ ions.^[^ ^39^ ^]^

## 3 The Significance of Transport Properties and Size Effects

Through the discussion in the previous chapters, it is evident that for potassium electrode materials, the primary challenges are the inadequate transport kinetics of potassium ions within the electrode materials and the structural degradation caused by significant volume changes during (de)intercalation. The key to addressing this issue lies in enhancing transport properties, elucidating the size effect, and improving the electro–chemo–mechanical coupling characteristics. Notably, the approaches employed to improve coupling characteristics have many similarities and overlaps with those used to regulate transport properties and size effects, and they will be discussed together in the relevant chapters.

### 3.1 Transport Properties and Size Effects

The transport properties of electrode materials (i.e., electronic conductivity, ionic conductivity, and diffusion coefficient) are fundamental and important. Understanding and enhancing these characteristics is crucial for improving electrochemical performance. Specifically, in the case of potassium‐ion batteries, which have a larger ionic radius and higher molecular weight, it is generally believed that their transport properties should be inferior to those of Li‐ion and Na‐ion batteries. Therefore, improving the transport characteristics of K‐ion batteries poses greater difficulty and importance. At the same time, the size effect will manifest in distinct characteristics as well.^[^ ^40^ ^]^

From the morphology/size perspective of electrode materials, the current mainstream electrode materials consist of micron‐, nano‐sized, and micro–nano composite materials. Initially, electrode materials primarily comprised micron‐sized materials, such as LiCoO_2_, which had an impact on their electrochemical performance due to their intrinsic transport characteristics. Afterward, the introduction of LiFePO_4_ led to the widespread utilization of nanoscale electrode materials in order to address the issues of low intrinsic ionic/electronic conductivities in these materials.^[^ ^41^, ^42^ ^]^ The development of nanosized LiFePO_4_ electrodes has resulted in significant thermodynamic,^[^ ^43^ ^]^ kinetic, and lithium storage mechanism changes^[^ ^44^ ^]^ for the materials, which have obtained considerable attention toward the size‐dependent effects. Simultaneously, with a better understanding of size effects, they have further influenced material modification and application processes, ultimately leading to successful commercialization.

From the perspective of electrode material modification strategies, the most commonly employed methods include doping, coating, and nanosizing. Among these approaches, doping can simultaneously regulate both ionic/electronic conductivity and diffusion coefficient; however, its impact on these conductivities may not be consistent.^[^ ^45^ ^]^ The frequently used coating method involves applying a layer of an electronically conductive phase, such as carbon coating, significantly enhancing the electronic conductivity of materials. This approach can be combined with doping together to control the transport properties of electrode materials. Additionally, if fast ion conductor materials are selected for coating purposes, it is possible to regulate the ionic conductivity of electrode materials as well and thereby improve the rate performance.^[^ ^46^ ^]^ The nanosizing strategy mainly regulates the transport properties, reducing electronic and ionic migration path lengths, thereby enhancing high‐rate performance. In most cases, due to their square relationship with size according to $\tau^{\delta }∼L^{2}/D_{Li}^{\delta }$ (where *L* is the particle radius or half‐thickness, $D_{Li}^{\delta }$ is the effective chemical diffusion coefficient of neutral lithium), this method yields immediate effects. Assuming the diffusion coefficient of an electrode material is 10^−12^ cm^2^ s^−1^, when the particle size is 100 microns, the ion diffusion time would be ≈3 years, comparable to the duration of a PhD program; if the particle size is reduced to 10 nm, the diffusion time decreases to merely one second, demonstrating a significant improvement. In summary, doping, coating and nanosizing modification methods can be adjusted to the transport characteristics, among which nanosizing is a direct and practical application of size effects, and it is important in improving the electro–chemo–mechanical behaviors.

**Figure 2:**
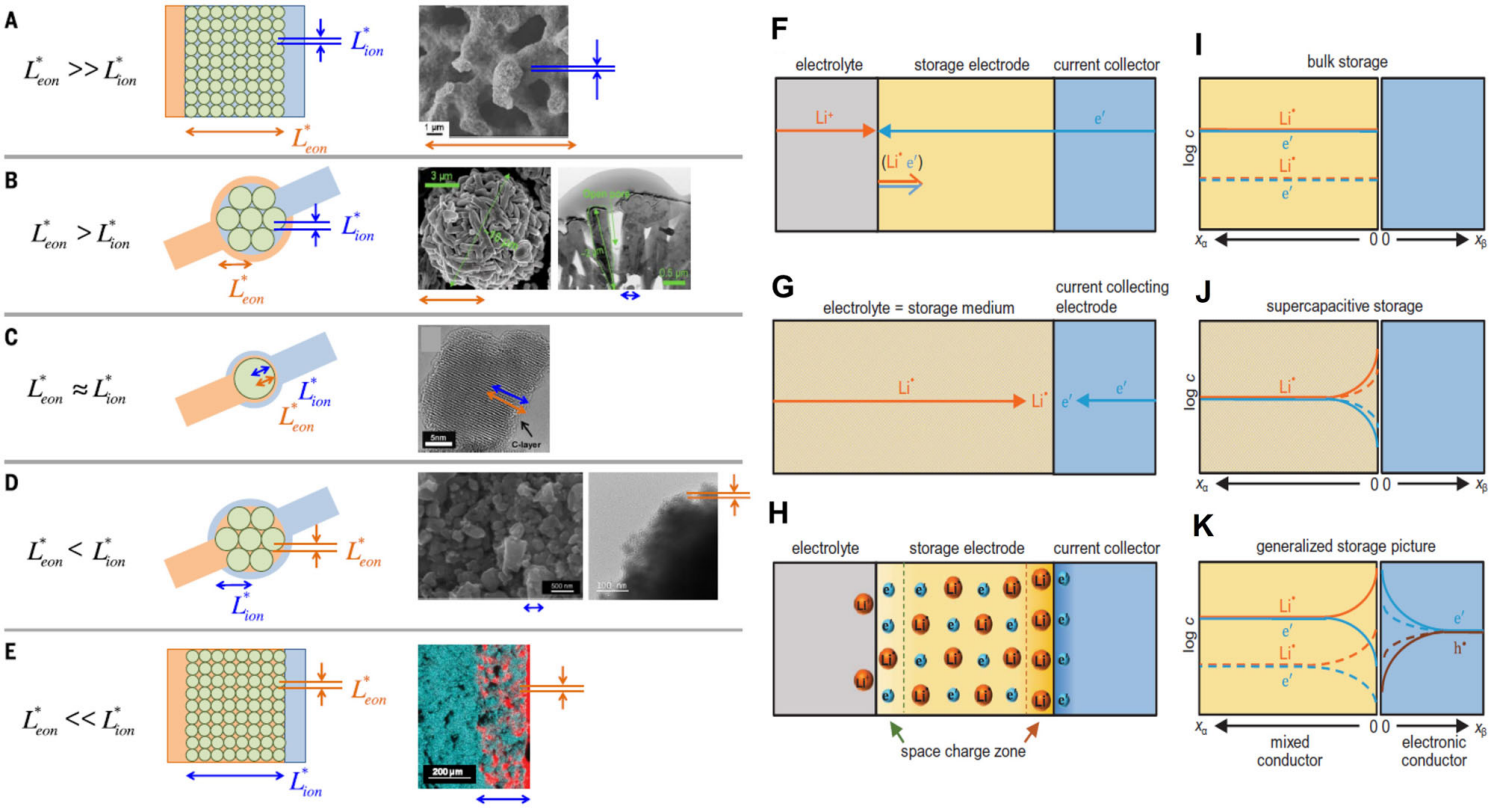
Nanoscale electrochemical circuitry design concepts based on different transport characteristics. A) porous C monolith; B) LiNi_0.5_Mn_0.5_O_2_ /Li_2_MnO_3_/C; C) LiMn_2_O_4_/C; D) Na_3_V_2_(PO_4_)_3_ /C; E) Li_10_GeP_2_S_12_/C). Reproduced with permission.^[^ ^47^ ^]^ Copyright 2017, AAAS.The concurrent occurrence of two processes in bulk‐phase storage, supercapacitor storage, and hybrid conductors, along with the corresponding charge‐carrier distribution diagram (F–K). Reproduced with permission.^[^ ^48^ ^]^ Copyright 2024, AAAS.

The presence of various electrode materials necessitates the design of electrochemical circuits that align with their distinct characteristics, posing a significant challenge. The fundamental approach involves leveraging the inherent transport properties of diverse materials to facilitate targeted design of nanoscale electrochemical conductive networks, thereby encompassing comprehensive utilization of transport properties and size effects. Although nanosizing can have an immediate impact, it is important to consider that its utilization may lead to increased material preparation costs and possible negative consequences, such as formation of SEI, impurity and agglomeration due to the increased interfacial reactivity by nano materials. Therefore, different transport properties and design concepts should be taken into account. As suggested by our previous paper,^[^ ^47^ ^]^ the optimal approach is to synthesize particles with the maximum size that enables complete discharge within the shortest duration and at the fastest rate. The shortest discharge time is defined as τ*. The corresponding particle radius or half‐thickness L* is defined as: $L^{*}=\sqrt{\alpha D^{\delta }\tau^{*}}$, where the constant α depend on charging mode and geometry. Since σ_*ion*_ and σ_*eon*_ (ionic conductivity and electronic conductivity) for electrode materials can be varied by orders of magnitude, resulting in varied optimal “wiring lengths,” $L_{ion}^{*}$ and $L_{eon}^{*}$, determined by $L_{ion}^{*}\approx L^{*}/\sqrt{t_{eon}}$ and $L_{eon}^{*}\approx L^{*}/\sqrt{t_{ion}}$ (*t_eon_* and *t_ion_* are the electronic and ionic transference numbers). The most cost‐effective approach to network design involves the following considerations: the electrode particle should be connected by the ionic conducting and electronic conducting phases, while ensuring that the wiring lengths for ions and electrons are kept close to $L_{ion}^{*}$ and $L_{eon}^{*}$, respectively. According to σ_*ion*_ and σ_*eon*_, it can be divided into five different cases.^[^ ^47^ ^]^ As shown in **Figure** **2**A–E, the electrode materials with different intrinsic transport properties lead to different preparing strategy for nanoscale electrochemical circuit constructing.

In addition, apart from the design of electrode materials, we can effectively manipulate the storage mechanism of ions by controlling their transport characteristics and size. From a storage mechanism perspective, the most common ones include bulk intercalation storage and supercapacitor interface storage. Considering transport properties, if electronic conductivity is negligible, it will exhibit an interface energy storage similar to that of a supercapacitor; otherwise, bulk intercalation storage and interface storage occur simultaneously, and the ratio depends on material size and voltage. Changes in size and adjacent phase properties are decisive factors in regulating the ratio and consequently shifting the energy storage mode between intercalation and interface mechanisms. The preference for intercalation mode is favored by thick samples or high electron energy collectors, while the supercapacitor mode is favored by thin samples or collectors capable of accommodating excess electrons. Therefore, adjusting the size and transport characteristics of the collector allows control over both energy density and power density of batteries (Figure 2F–K).^[^ ^48^ ^]^

### 3.2 Electro–Chemo–Mechanical Coupling Behaviors

Due to the significantly larger ionic radius of K^+^ compared to that of Li^+^ and Na^+^, greater lattice expansion occurs during K^+^ insertion into electrode materials. This expansion leads to more pronounced volumetric changes and local stress concentration. The dynamic accumulation and subsequent relaxation of strain energy have considerable effects on electrochemical performance. For example, Wang et al. employed finite element simulations to investigate both the overall and cross‐sectional stress distributions in carbon anodes after potassium ion insertion. In the case of the solid hexagonal prism (SHP) structure, stress concentration increases progressively from the interior toward the exterior, resulting in a distinct high‐stress region on the surface, where the maximum stress reaches 33.7 MPa. In contrast, for the hollow hexagonal prism with a wall of porous hollow tube (HHPPHT), the maximum stress is reduced to 22.5 MPa, with stress concentration primarily observed around the pores.^[^ ^49^ ^]^ Therefore, the HHPPHT structure effectively facilitates stress relaxation, thereby significantly enhancing the cycling stability of the electrode.

For potassium‐based layered oxide cathodes, the insertion and extraction of large K^+^ ions can also induce significant structural stress, leading to structural damage and performance degradation of the cathode materials. Zhou et al. reported that the synthesized peanut‐shaped hierarchical P3‐type K_0.45_Mn_0.5_Co_0.5_O_2_ (p‐KMCO) cathode, due to its unique hierarchical architecture, effectively mitigates mechanical deterioration caused by stress accumulation and maintains structural integrity during repeated cycling.^[^ ^50^ ^]^ After 150 cycles, the p‐KMCO cathode retained its original morphology, whereas the microstructure of the conventionally synthesized KMCO exhibited substantial damage. Furthermore, XRD analysis revealed that p‐KMCO underwent only minor peak shifts during charge–discharge processes without distinct phase transitions, indicating a single‐phase solid‐solution reaction mechanism. This further suggests that the structural strain is small and reversible, thereby contributing to enhanced long‐term cycling stability.

In addition to affecting individual particles within potassium electrodes, dynamic stress accumulation and release also play a significant role in multiparticle systems at the electrode sheet level. Liu et al. synthesized dual‐heteroatom doped mesoporous carbon sphere (DMCS) with unique radial pore channels and explored the stress regulation mechanism using finite element simulations.^[^ ^51^ ^]^ In single‐particle systems, the maximum stress generated during the K^+^ insertion of conventional carbon spheres (CS) reached 1.35 GPa, primarily concentrated on the outer surface, making them prone to fracture. In contrast, the mesoporous architecture of DMCS particles facilitated stress dispersion near the radial channels, reducing the peak stress to 0.74 GPa. For multiparticle systems, stress was concentrated in the contact areas between conventional CS particles (2.73 GPa), leading to electrode detachment; while the stress between DMCS particles was significantly reduced (1.29 GPa), maintaining better structural integrity. The mesoporous structure reduces the Young's modulus of the material, provides space for volume expansion, makes the distribution of K^+^ more uniform, and reduces stress accumulation. Therefore, the development of an effective stress‐buffering mechanism is essential for the rational design and feasible applications of potassium electrodes.

In addition, stress can also significantly affect ion diffusion kinetics, phase stability, and the ion storage mechanism, with particular attention needed for larger K^+^. With regard to the coupling between diffusion barriers and stress, Li et al. developed a strain‐relaxed potassium anode material, consisting of Bi/Bi_2_O_3_ nanodots embedded in amorphous carbon sheets (Bi/Bi_2_O_3_ NDs@CSs).^[^ ^52^ ^]^ This structure effectively mitigated volume expansion through the conversion reaction of Bi_2_O_3_. In situ TEM observations demonstrated that the anode underwent a 131.7% volumetric expansion during potassiation without experiencing structural fracture. Finite element simulations revealed a more uniform stress distribution within the Bi/ Bi_2_O_3_ NDs@CSs during this process, whereas bulk bismuth exhibited significant stress concentration. The improved stress distribution was attributed to the mechanical confinement provided by the carbon sheets and the buffering capacity of Bi_2_O_3_, both of which reduced structural degradation caused by localized stress and facilitated stable K^+^ diffusion pathways. Furthermore, simulation results indicated that the uniform distribution and rapid diffusion of K^+^ were closely associated with the mechanical stress state, with a lower stress environment being beneficial for maintaining high diffusion kinetics.

For potassium electrodes, the intrinsic physicochemical properties can also be modulated by stress/strain. Albina et al. investigated the electronic and magnetic characteristics of Prussian blue analogs (PBAs) K_2_Co[Fe(CN)_6_], under varying strain conditions using density functional theory (DFT) calculations.^[^ ^53^ ^]^ The results indicated that the tetragonal phase is more stable than the cubic phase, and a monoclinic phase may exist; strain can induce the material to transform from a semimetallic to a semiconducting state and affect the magnetic moment. These findings reveal the regulatory effect of strain on PBAs and further demonstrate the significance of the electro–chemo–mechanical coupling characteristics in the design of potassium‐ion battery electrodes.

By regulating the stress/strain characteristics, the storage mechanism of potassium ions can also be effectively controlled. Chong et al. synthesized a KFe_2/3_Mn_1/6_Cu_1/6_HCF cathode material using a medium‐entropy strategy.^[^ ^54^ ^]^ The high electronegativity of Cu facilitates the formation of strong Cu−N bonds, which enhances the structural rigidity and improves resistance to stress‐induced deformation. The entropy stabilization effect arising from the Mn−Fe−Cu ternary composition suppresses detrimental phase transitions. Additionally, the increased disorder at N‐coordination sites improves the lattice tolerance to volumetric changes, thereby mitigating structural stress. Therefore, the potassium ion storage of this material follows a zero‐stress solid solution reaction mechanism. During charge and discharge cycles, the material retains a cubic phase structure without undergoing monoclinic‐to‐cubic phase transitions, exhibiting minimal variations in lattice parameters and a volume change of only 0.7%. This greatly reduces the structural stress induced by repeated K^+^ (de)intercalation, allowing the material to preserve its structural integrity during cycling. In contrast, undoped KMnHCF experiences a monoclinic‐to‐cubic phase transition during K^+^ insertion/extraction, accompanied by a volume contraction of 1.6%. This significant volume change generates considerable stress, leading to structural damage and a decline in cycling performance.

Although we have already had some understanding the significance of electro–chemo–mechanical coupling characteristics in potassium electrodes, but the quantitative analysis of electro–chemo–mechanical coupling effects is still a challenging topic in this field. Of course, the regulation of transport properties and size effects, as previously discussed, represents an effective strategy for controlling such coupling properties. The regulation of such coupling behaviors in potassium electrodes can be achieved via component–structure control, nanostructure design, and interface engineering. These strategies correspond to the regulations of transport properties and size effects, which will be elaborated upon systematically in the relevant chapters.

## 4 Transport Properties in Potassium Electrodes

### 4.1 Characteristics and Fundamental Prerequisites of Potassium Ion Transport in Electrodes

Due to the higher molecular weight and the larger ionic radius of K^+^, their transport properties in electrode materials are generally affected, resulting in inferior transport characteristics compared to lithium or sodium ions within the same family. Taking the lithium cathode material as an example, the typical crystal structure includes layered structure, spinel structure and olivine structure, which correspond to the 2D, 3D, and 1D transport paths of lithium ions respectively. For layered cathode materials, LiCoO_2_ is the earliest commercially used lithium cathode material with excellent volumetric energy density and has been widely adopted in 3C electronic products as the most successful cathode material thus far. However, Na_x_CoO2 and K_x_CoO2 counterparts exhibit certain electrochemical activity but fail to provide satisfactory capacity and stability due to transport issues and structural changes caused by larger ionic radii.^[^ ^55^ ^]^

**Figure 3:**
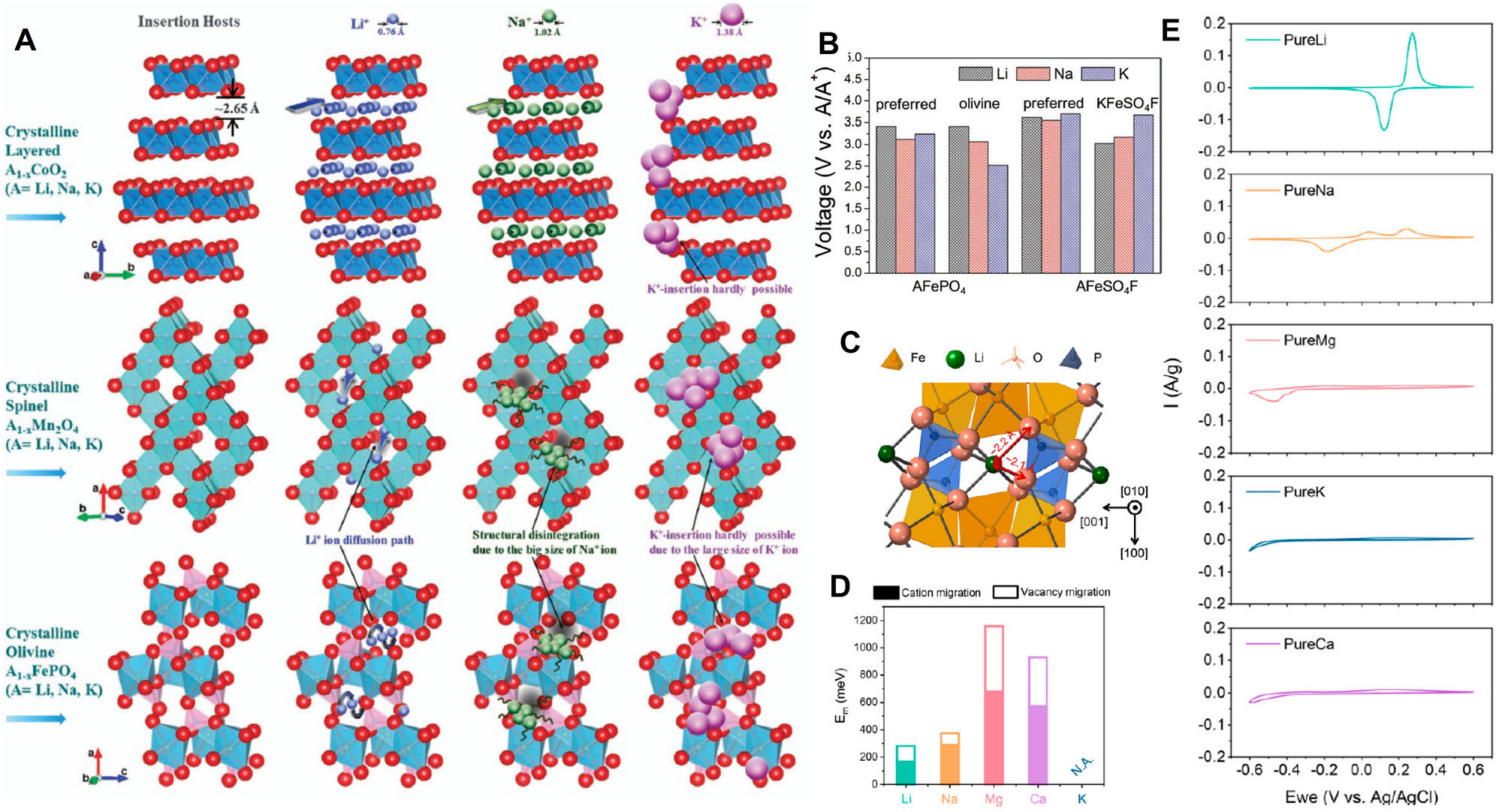
A) Schematic representation of Li^+^/Na^+^/K^+^ incorporated within various electrode architectures (layered, spinel, olivine structure). Reproduced with permission.^[^ ^56^ ^]^ Copyright 2014, Springer Nature. B) Calculated average voltage for AFePO_4_ and AFeSO_4_F (A = Li, Na, K). Reproduced with permission.^[^ ^6^ ^]^ Copyright 2018, Wiley‐VCH. C) The crystal structure of LiFePO_4_. D) Migration barriers for various cations by DFT calculations. E) CV measurements for olivine FePO_4_ in aqueous solution for various ions at a 0.03 mV s^−1^ scan rate. Reproduced with permission.^[^ ^57^ ^]^ Copyright 2022, PNAS.

In terms of cathodes with spinel structure and olivine structure, sodium ions can be inserted, but the transport properties become worse, and larger sodium ion intercalation will cause irreversible changes in the structure. For larger potassium ions, such materials are even unable to accommodate potassium ions effectively (**Figure** **3**A).^[^ ^56^ ^]^ As for the olivine LiFePO_4_ shown in the figure below (Figure 3C,D),^[^ ^57^ ^]^ it demonstrates different migration energy barriers for various ions such as Li^+^, Na^+^, K^+^, Mg^2+^, Ca^2+^. Although there is no reported migration energy barrier for potassium ions yet, it can be inferred that it will exceed those of Na^+^, Li^+^, and even bivalent Mg^2+^. Additionally, with the gradual increase in radius of Li^+^, Na^+^ and K^+^ ions, the transport characteristics gradually deteriorate, and it is found through calculation that the insertion voltage of various ions also shows a gradual decline (as shown in the Figure 3B).^[^ ^6^ ^]^ Furthermore, due to variations in size and transport properties among different ions, significant differences are observed in their final electrochemical properties (as illustrated in Figure 3E).^[^ ^57^ ^]^ The intercalation of K^+^, Na^+^, Li^+^ and even Ca^2+^, Mg^2+^into olivine FePO_4_ hosts are compared by cyclic voltammetry (CV) results in 1 m aqueous cation solutions (scan rate: 0.03 mV s^−1^). For the lithium insertion (FePO_4_ in 1 m LiCl), the CV curve exhibits a pair of symmetric cathodic and anodic peaks, at its thermodynamic value (0.213 V vs Ag/AgCl), which also exhibits good reversibility. As far as for Na^+^, two anodic current peaks are found due to the Na_0.7_FePO4 intermediate phase formation during the desodiation process, while only one cathodic current peak is found for sodiation process (1 m NaCl). The value of the current peak for Li^+^ insertion/extraction is almost more than 3 times higher than that for Na^+^ at the same scan rate, demonstrating slower of Na^+^ transport and storage compared to Li^+^ in olivine FePO_4_ hosts. However, there are no redox peaks for K^+^ insertion/extraction in 1 m KCl solutions, which demonstrates that K^+^ will not (de)intercalate in olivine FePO_4_ due to the larger ionic radii and migration barriers of K^+^.

Therefore, when selecting and designing potassium electrode materials, preference should be given to materials with larger ion transport channels (1D, 3D) or materials with greater layer spacing (2D), taking into account the relatively larger size of potassium ions.^[^ ^58^, ^59^, ^60^ ^]^ Zhang et al. tested a series of layered vanadates for potassium electrodes in terms of (d_100_), i.e., interlayer distance between planes of adjacent (100), which is the key factor for the potassium transport and the electrochemical performance.^[^ ^61^ ^]^ (NH_4_)_0.5_V_2_O_5_ with a 9.52 Å d_100_ exhibits a higher capacity and faster K^+^ diffusion than NH_4_V_3_O_8_ with a 7.80 Å d_100_, indicating the significance of interlayer distance for K^+^ transport in electrodes. They also found that K^+^ preintercalation into V–O slabs can further stabilize the vanadates structure, which results in higher electrochemical cycling stability.

Increasing the layer spacing or widening the potassium ion transport channel can not only enhance transport properties but also serve as an effective strategy to address electro–chemo–mechanical coupling issues, thereby mitigating volume expansion and alleviating stress more effectively. For example, Guo's group utilized the short‐range ordered structure of amorphous carbon to expand the spacing of the layer to 0.52 nm, surpassing the layer spacing of traditional graphite. This approach significantly reduces volume expansion during cycling and achieves superior cyclic stability.^[^ ^62^ ^]^

### 4.2 Cases of Non‐Ion Size‐Dependent Transport Anomalies

As discussed in previous chapters, larger K^+^ result in poorer transport characteristics compared to Li^+^ and Na^+^. Therefore, the transport of K^+^ is closely related to the sizes of ion channels, and electrodes with larger ion channels should be preferred. However, the transport properties are not solely determined by the size of the intercalation ion or the channel dimensions. A smaller size of ion is not always advantageous. Both potassium cathode materials and anode materials can exhibit anomalous transport phenomena under certain conditions. When considering transport characteristics, appropriate frame sizes of host materials, interactions of insertion ions and host materials, and a synergy between thermodynamics and dynamics significantly impact the material's final transport properties.

**Figure 4:**
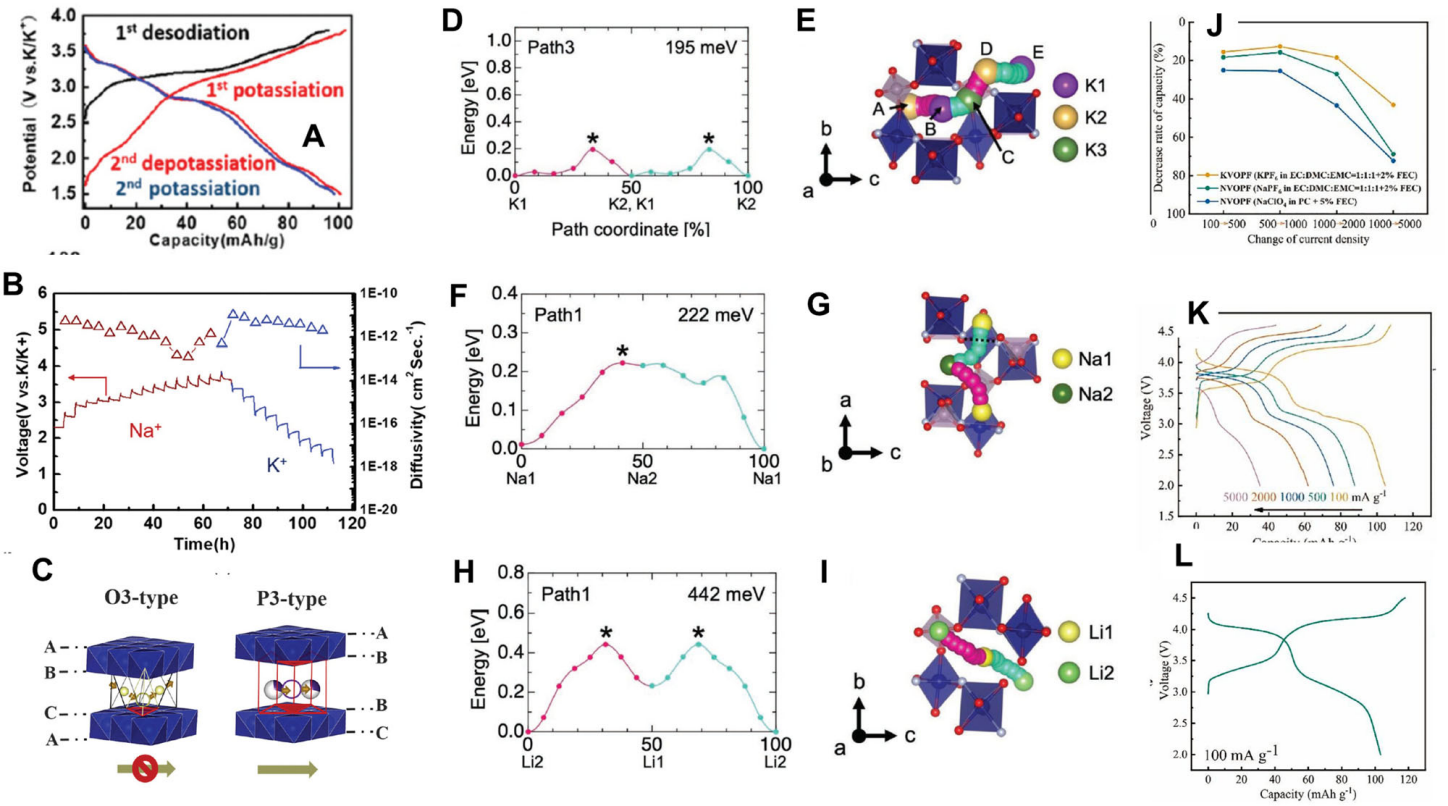
A) Charge–discharge curves of NCRO for K storage. B) The diffusivity of NCRO calculated by GITT. C) Schematic illustration displaying indirect Na ion (yellow balls) and direct K ion (white/purple balls) diffusion paths between the layered structure of O3 and P3. Reproduced with permission.^[^ ^63^ ^]^ Copyright 2019, RSC. D) Migration barrier for K^+^ in K_x_VPO_4_F and E) Migration path for K^+^; F) Migration barrier for Na^+^ in Na_x_VPO_4_F and G) Migration path for Na^+^; H) Migration barrier for Li^+^ in Li_x_VPO_4_F and I) Migration path for Li^+^. Reproduced with permission.^[^ ^64^ ^]^ Copyright 2019, Wiley‐VCH. J) Comparison of rate performance of KVOPF and NVOPF in different electrolytes. K) Charge and discharge profiles of KVOPF. L) The first charge–discharge profiles of KVOPF/hard carbon full cell. Reproduced with permission.^[^ ^65^ ^]^ Copyright 2022, Elsevier.

Different intercalation ions in the same host electrode may have different transport paths, which will lead to various and anomalous transport characteristics. Layered oxide Na_0.9_Cr_0.9_Ru_0.1_O_2_ (NCRO) can be a suitable host for both sodium and potassium storage, and KCRO can be obtained by electrochemical ion‐exchange. Zhou et al. applied the galvanostatic intermittent titration technique (GITT) technique to evaluate the chemical diffusion coefficient of Na^+^ and K^+^, respectively, in this layered oxide (**Figure** **4**A,B).^[^ ^63^ ^]^ In spite of larger K^+^, the calculated chemical diffusion coefficient of K^+^ is in the order of 10^−13^–10^−12^ cm^2^s^−1^, which is comparable and even higher than that of sodium, whose values are between 10^−14^ and 10^−12^ cm^2^s^−1^. The transport anomaly may arise from the disparity in transport pathways resulting from the discrepancy in ionic radii between sodium and potassium ions. During the charge process, a phase transition (O3 → P’3) occurs. Na^+^ transport paths are O (octahedral sites)‐T (tetrahedral interstitial sites)‐O (octahedral sites), which requires the assistance of tetrahedral interstitial sites to accomplish the transport. However, in the O‐type structure, such ionic migration occurs through a non‐direct pathway, which requires to overcome of elevated activation energies for interlayer species transport. On the other hand, during K^+^ insertion, the diffusion path is from prism sites to prism sites, which is a direct diffusion, and the phase transition is extremely weak as well (Figure 4C). Finally, the improved transport properties of K^+^ lead to better performance. The capacity is 100.6 mAh g^−1^, maintaining 80.1% retention after 300 cycles at 0.5C and 81.2% retention through 500 cycles at 5C.

In addition, the large alkali‐ion migration channel for the transport ion is not always the best choice. A typical example here is K_x_VPO_4_F (x ≈ 0) polyanionic cathode, which can work as a versatile and stable host for Li^+^, Na^+^, and K^+^.^[^ ^64^ ^]^ Its unique crystalline structure with the large cavity size enable the intercalation of alkali ions, and even fast migration of them. For the nudged elastic band (NEB) calculations (Figure 4D–I), it exhibits 195, 222, and 442 meV in migration barriers for K*_x_* VPO_4_F, Na*_x_* VPO_4_F, and Li*_x_* VPO4F, respectively are observed. Interestingly, the kinetics of Li insertion in K*_x_* VPO_4_F (*x ≈* 0) is even worse than K and Na, and K exhibits the lowest migration barrier in such a host. Therefore, even in the same host, different alkali ions with various sizes could have different transport paths and transition states due to the different site occupancies. The large migration channels are not always the best choice for fast transport properties, and it is significant to tailor the void space and transition states in terms of specific intercalation ions for the host. The same discovery is found in another polyanionic cathode material K_3_V_2_O_2_(PO_4_)_2_F, for potassium ion batteries.^[^ ^65^ ^]^ The migration barriers for K^+^ are comparable or even lower than those of Na^+^, which is confirmed by the density functional theory (DFT) calculations. Due to the excellent transport properties of K^+^, a micron‐sized carbon‐free K_3_V_2_O_2_(PO_4_)_2_F cathodes can be successfully prepared, whose rate performance is even better than its sodium counterpart, as shown in Figure 4J. This potassium cathode displays the high capacities of 105, 62, 35 mAh g^−1^ at 100, 2000, 5000 mAg^−1^ (Figure 4K). For K_3_V_2_O_2_(PO_4_)_2_F/hard carbon full cell (Figure 4L), it shows a high capacity of 93.5 mAhg^−1^ after 50 cycles, indicating the potential for the application for such micron‐sized cathodes.

**Figure 5:**
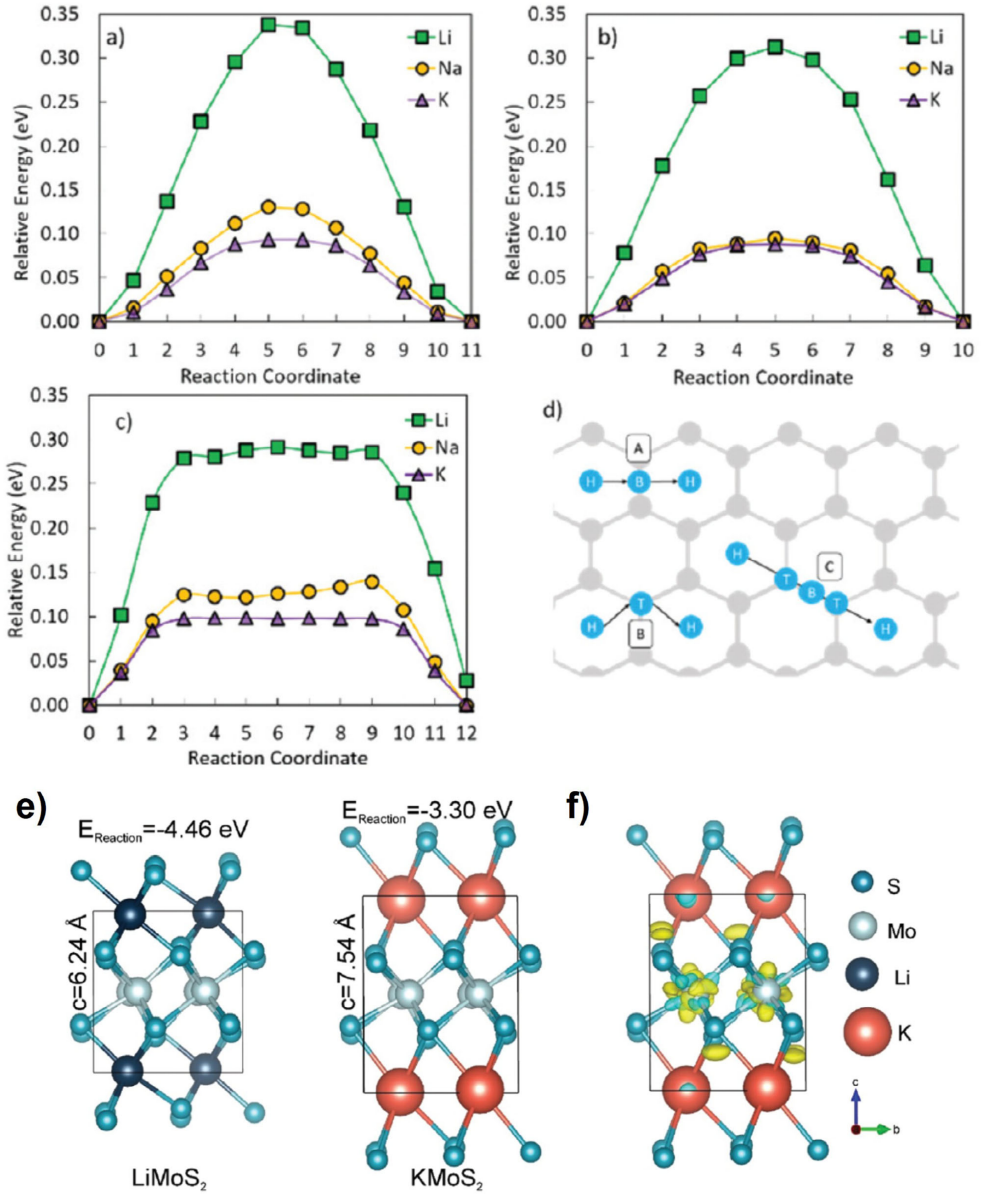
Schematic of the three metal diffusion paths (A–C in (d)) on graphene, and their respective migration barriers for Li, Na, and K on graphene (a) H–B–H, (b) H–T–H, and (c) H–T–B–T–H using the CI‐NEB method. Reproduced with permission.^[^ ^67^ ^]^ Copyright 2019, RSC. (e) The calculated structures of KMoS_2_ and LiMoS_2_; (f) Charge density difference distributions of KMoS_2_. Reproduced with permission.^[^ ^68^ ^]^ Copyright 2020, RSC.

Carbon materials are important anodes for Li‐, Na‐, and K‐ion batteries, exhibiting distinctive transport anomalies in such materials as well.^[^ ^66^ ^]^ Graphite is the most stable and successful anode for Li‐ion batteries. During Li^+^ intercalation, the graphite can form graphite intercalation compounds (GICs), showing a theoretical capacity of 372 mAh g^−1^ with the formation of LiC_6_. However, for the Na^+^ and K^+^, the storage behaviors are different. Aa for larger Na^+^, it can hardly intercalate in the interlayers of graphite, and can not form stable Na–C compounds. By contrast, in spite the larger size of K^+^ versus Na^+^, K^+^ can intercalate in the graphite forming K‐GIC, such as KC_36_, KC_24_, and KC_8_, at low working potential, finally exhibiting a high capacity (∼273 mAhg^−1^).^[^ ^66^ ^]^ For the hard carbon anodes, although they are efficient anodes for storage of Na^+^ and K^+^, the different and anomalous transport properties are observed. In the hard carbon, K^+^ demonstrates superior ionic diffusivity compared to Na^+^, a phenomenon originating from different binding energies (K–C vs Na–C) coupled with potassium's reduced charge density.^[^ ^66^ ^]^ For the graphene anodes, adsorption/desorption behavior determines the transport properties for alkali ions on the graphene surface. In order to evaluate the different migrations on the surface of graphene among various alkali ions, the climbing image nudged elastic band method (CI‐NEB) was applied.^[^ ^67^ ^]^ As shown in **Figure** **5**a–d, there are three different diffusion paths to be investigated. Li exhibits the highest diffusion barriers in all three diffusion paths, while K shows the lowest migration barriers, and that of Na is in between. Their results indicate that K^+^ transports more easily on the graphene surface compared to the Na and Li counterparts, in spite with larger size of K. In addition, for few‐layered graphene, the transport of K^+^ includes both intercalation into the interlayers and adsorption on the graphene surface; as for Na‐ion batteries, Na^+^ can only transport on the surface, since it is very difficult to insert into the few‐layered graphene's interlayers.

Moreover, the thermodynamics and dynamics of Li^+^, Na^+^, and K^+^ transport are affected by the varying sizes of different ions, which in turn can alter their storage mechanism and subsequently impact their transport characteristics. For example, MoS_2_ as a model electrode is investigated to evaluate the transport properties and electrochemical performance.^[^ ^68^ ^]^ Although K^+^ has a larger size, intercalation of K^+^ in MoS_2_ exhibits unusual and special electrochemical and structural stability compared to Li^+^ and Na^+^. The enthalpies of conversion reactions of the MoS_2_‐intercalated compounds into Mo and alkali sulfide are −4.46, −3.16, and −3.30 eV f.u.^−1^ for Li, Na, and K, respectively (Figure 5e,f). This demonstrates that thermodynamically, the driving force of the conversion reaction for K is smaller, resulting in the possibility of preservation of the layered structure, where intercalation occurs instead of the conversion reaction. Indeed, the intercalated compounds are found in the experiments, and this indicates that the kinetics factor plays a significant role as well. From the charge distributions of KMoS_2_, charges accumulated between K and S atoms are observed, indicating the interaction or bonding between them. In short, the conversion reaction of K‐MoS_2_ systems is thermodynamically and kinetically suppressed.

### 4.3 Regulation of Intrinsic Transport Properties

The regulation of transport characteristics can be categorized into intrinsic and extrinsic regulation. The most commonly employed non‐intrinsic regulation method is the size control, which will be extensively discussed in the section on size effects. This section primarily focuses on the aspects of intrinsic regulation.

#### 4.3.1 Coating

In principle, depending on the properties of the coating materials, such as whether it is an ionic conductor or an electronic conductor, the coating can enhance the corresponding ionic or electronic conductivity effectively. From a practical application perspective, coating is considered to be the simplest and most effective method for improving electronic conductivity. The choice of coated material, thickness of the coating, and its uniformity all play crucial roles in determining the electron transport and the electrode performance. Among various coating materials, carbon‐based materials have demonstrated remarkable efficacy in enhancing electronic transport.^[^ ^69^, ^70^, ^71^, ^72^, ^73^, ^74^, ^75^ ^]^ For instance, Qiu et al. applied conductive polypyrrole to uniformly coat Prussian blue potassium cathode nano‐particles, which have an intrinsic poor electronic conductivity, leading to a higher rate capability.^[^ ^76^ ^]^ Concerning the coating, there are significant similarities with lithium and sodium electrodes that will not be elaborated upon here.

#### 4.3.2 Doping

An effective approach to improve the intrinsic K^+^ transport properties within electrodes is through doping.^[^ ^77^, ^78^, ^79^ ^]^ In most cases, coating is effective for the modification of electronic conductivity. Doping, however, allows for simultaneous modification of both ionic and electronic conductivities. It should be noted that this modification does not necessarily guarantee improvement for both ionic and electronic transport; hence, in practice, it is common to employ doping and coating technologies concurrently. In addition, doping not only modulates the transport characteristics but also improve the electro–chemo–mechanical coupling properties. For instance, doping can widen the potassium ion transport channel, suppress lattice distortion, thereby reducing volume expansion and stress, and consequently improving lattice stability and cycling stability.

For potassium cathodes based on an intercalation reaction, doping of transition metal element sites is the most common and effective modification method. Layered oxide is a promising potassium cathode owing to its high energy density. Nonetheless, the presence of large potassium ions intercalation in the lattice poses challenges such as poor transport characteristics and lattice instability that require urgent resolution. There is are great number of reports that transition‐metal doping with nickel, cobalt, and iron or a combination of them.^[^ ^80^, ^81^, ^82^, ^83^, ^84^ ^]^ For example, Selvan et al. prepared Co‐doped P3‐type K_0.67_MnO_2_ (optimized composition K_0.67_Mn_0.95_Co_0.05_O_2_), leading to an improved diffusion coefficient (10^−9^−10^−8^ cm^2^ s^−1^ by GITT analysis) and the minor structural phase transition, because of cooperative simultaneous redox activity with the Jahn−Teller Mn^3+/4+^ and overlapping of Co^3+^/^4+^ with O 2p orbitals.^[^ ^85^ ^]^ This clearly demonstrates the crucial significance of doping in modulating transport properties and electro–chemo–mechanical coupling characteristics.

**Figure 6:**
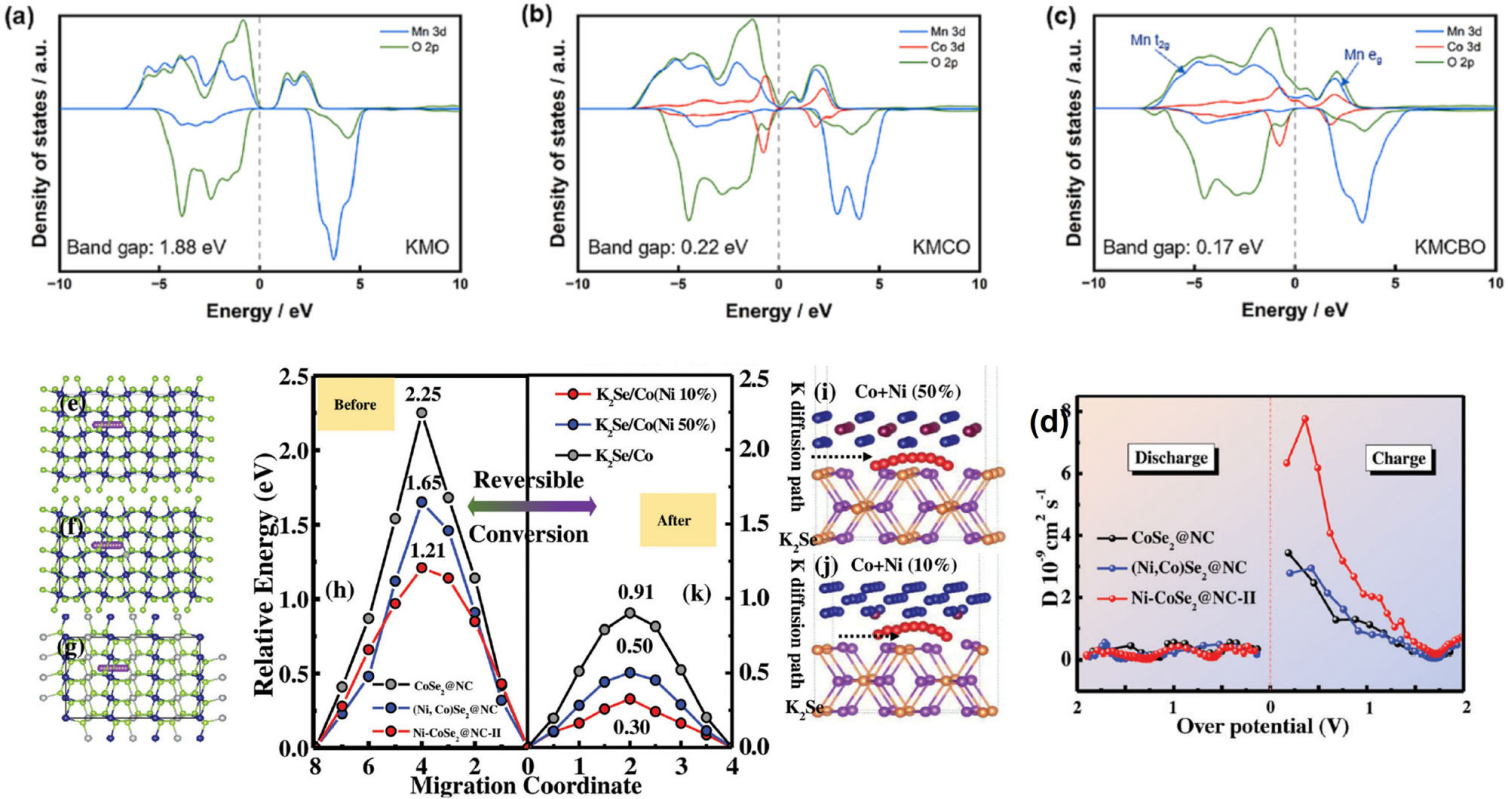
a–c) pDOS of transition metal 3d and O 2p orbitals in various samples. Reproduced with permission.^[^ ^86^ ^]^ Copyright 2023, Elsevier. d) Diffusion coefficients of CoSe_2_@NC, (Ni, Co)Se_2_@NC, and Ni‐CoSe_2_@NC‐II. e–g) Diffusion paths of K^+^ in the structure of CoSe_2_, Ni‐CoSe_2_, and (Ni, Co)Se_2_. i,j) The K_2_Se/Co(Ni) interface for (Ni, Co)Se_2_ and Ni‐CoSe. h,k) Energy barriers for K^+^ diffusion during the charge‐discharge procedure. Reproduced with permission.^[^ ^91^ ^]^ Copyright 2022, Wiley‐VCH.

In addition to doping of transition metal elements, the selection of appropriate nonmetallic elements for interstitial doping is also a viable strategy. Zhou et al. introduced small B^3+^ ions (0.27 Å ion radius) in the tetrahedral interstice in layered P3‐K_0.5_MnO_2_, together with Mn‐site partial doping with Co substitution to prepare a P3‐type K_0.5_Mn_0.8_Co_0.2_B_0.1_O_2_ (KMCBO).^[^ ^86^ ^]^ Due to the stronger B‐O covalent bonds (Mn‐O: 402 kJ mol^−1^ vs B‐O: 515 kJ mol^−1^), the transport properties and Jahn‐Teller distortion can be effectively improved by controlling neighboring O sites for K^+^ ions diffusion. The charge distribution and electronic structure of such materials are investigated by DFT calculations. After doping with Co and B, the band gap of this material is reduced from 1.88 to 0.22 and 0.17 eV (**Figure** **6**a–c), demonstrating enhanced electronic conductivity by Co and B doping. Moreover, with the help of B for stabilizing the crystalline structure, no irreversible phase transition is found in spite of the insertion of larger K^+^, leading to better transport properties and a good cycling performance.

In addition to cationic doping, anionic doping can also effectively regulate the transport characteristics. As previously mentioned, in principle, larger channels contribute to the enhancement of transport properties in potassium ion batteries, and anionic doping can play a role in expanding the transport channels. Fang et al. prepared K_0.6_CoO_2‐x_N_x_ porous nano‐frame by doping O with N, which can simultaneously enhance the ionic conductivity and electronic conductivity.^[^ ^87^ ^]^ As confirmed by experiments and calculations, partial doping of O by N can not only expand the interlayer spacing, accommodate larger K^+^, and improve transport of K^+^, but also increase the electronic conductivity, resulting in high‐performance potassium cathodes. The system demonstrates a capacity of 86 at 50 mA g^−1^ current density, with 77.3% cyclic stability preserved through 400 electrochemical cycles.

Compared to layered oxides, polyanionic potassium cathode materials exhibit superior thermal and structural stability; however, their intrinsic transport characteristics, particularly electronic conductivity, are inadequate and necessitate improvement through effective approaches such as doping. Komaba et al. synthesized MWCNT‐modified Mg‐doped KFe_1−x_Mg_x_SO_4_F (x = 0, 0.05, 0.1).^[^ ^88^ ^]^ Its orthorhombic structure can be retained with the lattice contraction, since the doping Mg^2+^ is smaller compared with Fe^2+^. After such doping, the optimized composition KFe_0.95_Mg_0.05_SO_4_F is confirmed, which can widen the diffusion channels for K^+^ and narrow the band gap. Finally, the system demonstrates a capacity of 118 mAh g^−1^ at 0.05C current density.

For carbon anodes, doping is an efficient means to enhance K^+^ transport as well. For carbon materials, nonmetallic doping is a more common approach, usually through one or more nonmetallic elements doping, introducing defects, thereby increasing the storage site of potassium ions, and promoting potassium ion transport. Alshareef et al. prepared nitrogen‐doped, graphitic nanocarbons (GNCs) with rich defects by heating a Ni‐EDTA coordination compound.^[^ ^89^ ^]^ Through such N‐doping, it increases the number of active sites for K^+^ storage and provides fast pathways for K^+^ transport. The optimized anode (noted as GNC600), which has nitrogen doping at a high level, leads to a high pseudocapacitive contribution. The short‐range ordered defect‐rich structure results in a high diffusion coefficient of potassium and high‐rate performance.

The transport properties of K^+^ can also be regulated by controlling defects. Xiong et al. applied a self‐template strategy to achieve carbon anodes with defect‐selectivity and order‐in‐disorder structure.^[^ ^90^ ^]^ The reversible carbon vacancy and other defect sites are identified, while the less reversible portions of heteroatomic defects are preferentially eliminated through gas release during pyrolysis. The intrinsic conductivity is enhanced by incorporating nano‐sized graphitic frameworks into defect‐rich regions. DFT calculation confirmed that the carbon‐vacancies had faster ion/electron transport kinetics and higher reversibility compared to heteroatom‐defects, and the nano‐graphitic network can improve the charge transfer kinetics by constructing a fast electron migration path. As a result, the potassium anode demonstrates a gravimetric capacity of 425 mAh g^−1^ at 0.05 A g^−1^, and it keeps 237.4 mAh g^−1^ even at 1 A g^−1^.

The potassium anode, based on the conversion reaction, can also be doped to increase the ion/electron transport kinetics. Nonetheless, the characteristics of multiphase reactions require us to pay attention to the transport properties of both the materials before the reaction and the multiphase coexistence after the reaction. Li et al. regulated the electronic structure and transport properties of CoSe_2_ by accurately regulating the Ni/Co ratio; the doped Ni‐CoSe_2_ has shown simultaneously enhanced ionic and electronic diffusion as well as electrochemical performance.^[^ ^91^ ^]^ As shown by DFT calculation, K migration paths in the doped and pristine samples are displayed in Figure 6e–g. Ni‐CoSe_2_ shows an obviously lower diffusion barrier of K (1.21 eV) than CoSe_2_ (2.25 eV) and (Ni, Co)Se_2_ (1.65 eV). A new interface formed between K_2_Se and Co (Ni) with significantly decreased K diffusion barriers after the conversion reaction (Figure 6k). Corresponding migration pathways of K at the heterointerface are displayed in Figure 6i,j. Moreover, the density of states (DOS) of CoSe_2_ and Ni doped CoSe_2_, it indicates the enhanced electronic conductivity. Finally, the doped anodes deliver a high capacity of 400 mAhg^−1^ after 100 cycles, and the capacity is maintained at 284 mAhg^−1^ even at a high rate of 2 Ag^−1^.

#### 4.3.3 High Entropy Electrodes

In addition to conventional doping, the recent emergence of multielement synergistic doping, known as high entropy doping, has obtained significant attention from researchers. High entropy doping has demonstrated its pivotal role in various electrode materials by enhancing cycling performance, regulating transport characteristics, and improving stability, etc.^[^ ^92^ ^]^ Currently, research on high entropy electrodes for K‐ion batteries is still in its nascent stage, with limited reports and the absence of relevant reviews. This chapter aims to provide a comprehensive summary of current high entropy electrodes for potassium ion batteries, focusing on their impact on potassium ion transport properties, electro–chemo–mechanical coupling characteristics, and other performance enhancements. From a few reported examples, for potassium ion batteries, high entropy electrodes can also play an important role, which is a promising direction we can focus on.

**Figure 7:**
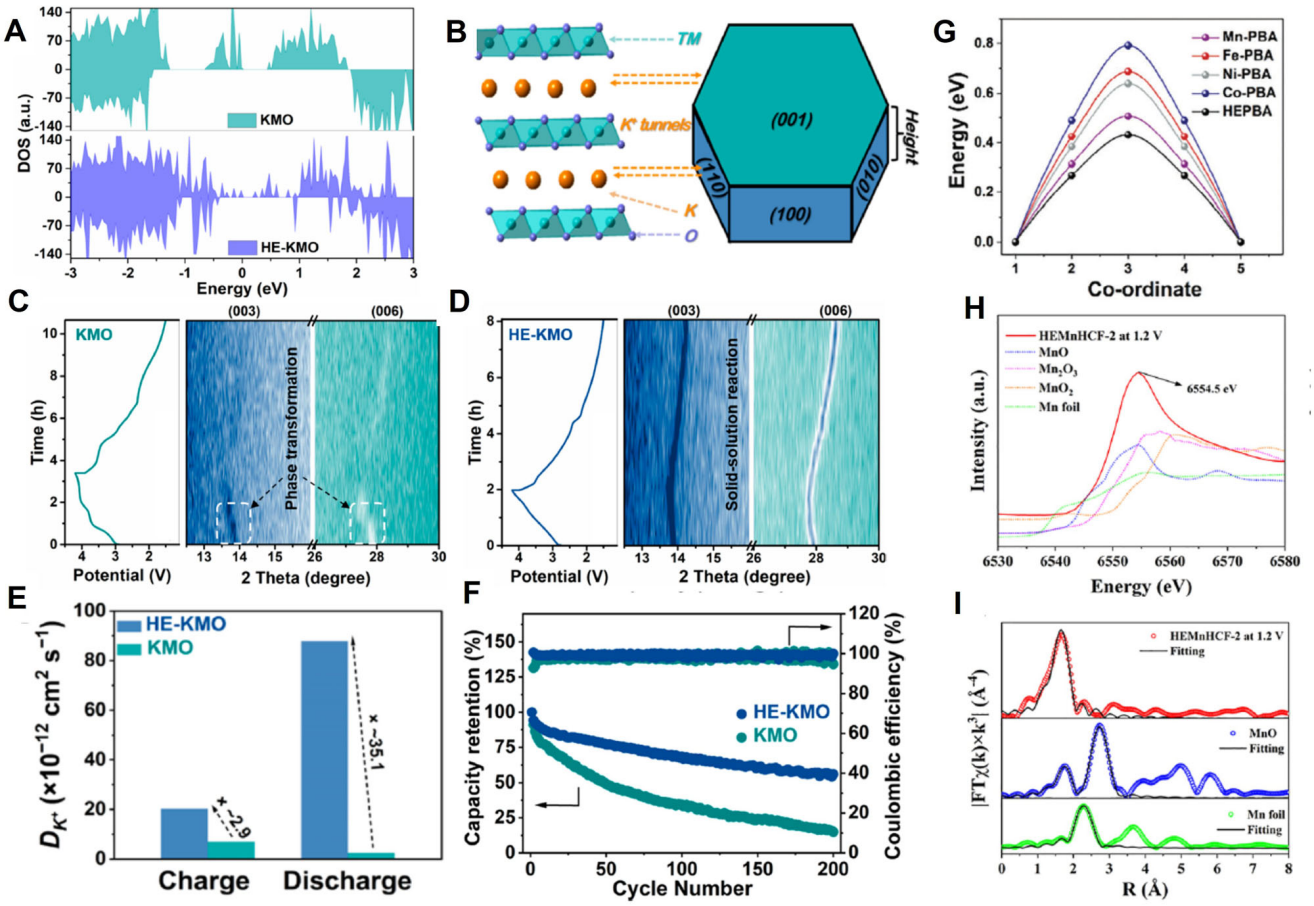
A) Total DOSs of KMO and HE‐KMO. B) Schematic illustration of the layered K_x_TMO_2_ structure. In situ XRD patterns of C) KMO and D) HE‐KMO electrodes. E) Diffusion coefficient of KMO and HE‐KMO in charge–discharge procedure. F) Cycle stability of He‐KMO and KMO. Reproduced with permission.^[^ ^93^ ^]^ Copyright 2023, ACS. G) The migration energy barriers of the K^+^ diffusion in M‐PBAs and HEPBA. Reproduced with permission.^[^ ^96^ ^]^ Copyright 2024, Wiley‐VCH. H) and I) XANES and EXAFS spectra at K‐edges of HEMnHCF‐2 at 1.2 V. Reproduced with permission.^[^ ^97^ ^]^ Copyright 2024, Elsevier.

The high‐entropy strategy was proposed for alloy design, i.e., high entropy alloys, which with five or even more metal elements. The scope of its application was later expanded to include battery materials, with recent advancements in reporting high entropy designs specifically for potassium ion batteries. The high entropy strategy is implemented in the layered oxide potassium cathodes, yielding remarkable outcomes. For example, Zhou et al. prepared layered cathodes K_0.45_Mn_0.60_Ni_0.075_Fe_0.075_Co_0.075_Ti_0.10_Cu_0.05_Mg_0.025_O_2_ (HE‐KMO) for potassium ion batteries by high‐entropy design concept.^[^ ^93^ ^]^ The transport properties including both electronic conductivity and ionic conductivity, as well as diffusion coefficient are highly improved by such a strategy. The high‐entropy electrode changes the band gap of the electrode material, leading to variations of the electronic conduction. As shown in **Figure** **7**A, it demonstrates that bandgap of HE‐KMO (spin‐up state‐0.19 eV; spin‐down state‐1.28 eV) is reduced compared to KMO (spin‐up state‐0.45 eV; spin‐down state‐3.38 eV) from the total density of states (DOS), leading to an increase in electronic conductivity.

The crystal morphology of the layered oxide potassium cathode will impact the exposure of various crystal facets, while the transport characteristics of potassium ions will vary across different crystal facets. The utilization of high entropy electrodes can influence the crystalline structure and thereby regulate ion transport. For example, for layered oxide, the [010] facets on the side provide open channels for K^+^ transport, whereas the [001] facets on the front remain inactive (Figure 7B). The preparation of high‐entropy cathodes with layered structure enables the adjustment of crystal facet energy, facilitating the growth of crystals into a robust brick‐like morphology along the [001] crystal orientation instead of a thin flake structure, which results in better K^+^ transport. Due to the increase in both electronic and ionic conductivity, the diffusion coefficient is increased as well. As calculated by the GITT, the corresponding diffusion coefficients of KMO and HE‐KMO electrodes are investigated. The average D values of HE‐KMO are 2.03 × 10^−11^ and 8.78 × 10^−11^ cm^2^s^−1^ for charge and discharge processes, which are higher than that of KMO (7.06 × 10^−12^ and 2.5 × 10^−12^ cm^2^s^−1^, respectively, for charge and discharge processes), demonstrating improved transport properties (Figure 7E). As a result, the improved rate capability is observed for HE‐KMO compared to KMO (39.6 vs 7.5 mAh g^−1^ at 5C).^[^ ^93^ ^]^

In addition to enhanced transport properties, a high entropy electrode also offers numerous other advantages, such as changing the phase transition mechanism and improving electro–chemo–mechanical coupling behaviors. From in situ X‐ray diffraction (XRD) (Figure 7C,D), for HE‐KMO, the diffraction pattern only exhibits a shift in peak position without the appearance of any new diffraction peaks throughout the entire charging process, demonstrating a solid‐solution single‐phase process (maintaining a P3 phase), which leads to excellent structural stability and reversibility. However, KMO exhibits inherent crystallographic distortions in its pristine state while concurrently displaying structural phase evolution during K^+^ deintercalation processes. Improved electro–chemo–mechanical coupling behaviors of HE‐KMO lead to a better cycling performance (Figure 7F), i.e., the capacity retention of HE‐KMO is 60.4% after 200 cycles, which is much higher than that of KMO (only 17.3%).^[^ ^93^ ^]^ In summary, the high‐entropy design can effectively inhibit the space charge ordering formation and enhance the covalent interaction between transition metal (TM) and oxygen (O), thereby suppressing the unfavorable phase transition as well as subsequent particle cracking and dissolution of TM during repeated electrochemical potassium‐ion insertion/extraction processes.

A similar high entropy strategy was also applied to potassium layered oxide with different high entropy components. Besides previously mentioned K_0.45_Mn_0.60_Ni_0.075_Fe_0.075_Co_0.075_Ti_0.10_Cu_0.05_Mg_0.025_O_2_, K_0.7_Fe_0.05_Co_0.1_Mn_0.75_Ni_0.05_V_0.05_O_2_,^[^ ^94^ ^]^ K_0.6_Ni_0.05_Fe_0.05_Mg_0.05_Ti_0.05_Mn_0.725_O2,^[^ ^95^ ^]^ K_0.27_Mn_0.9_ (Fe_0.02_Co_0.02_Ni_0.02_Zn_0.02_Mg_0.02_) O_2_ have also been prepared. The significance of the disorder structure introduced by the high entropy strategy in enhancing the transport characteristics and electrochemical properties is emphasized by all these investigations. However, the impact of varying potassium content and different doping elements on the layered oxides remains uncertain.

The high entropy strategy can also play an active role in the Prussian blue potassium cathode in terms of transport properties and other related characteristics. Zhu et al. prepared high‐entropy Prussian Blue analog (PBA) K_1.36_Mn_0.22_Fe_0.22_Ni_0.22_Mg_0.13_Co_0.21_[Fe(CN)_6_]_0.84_□_0.16_·1.62H_2_O for an aqueous K ion battery.^[^ ^96^ ^]^ As shown in Figure 7G, the high entropy effect leads to a lower migration barrier for the K^+^ diffusion in HE‐PBA compared to pristine PBA. The calculated diffusion coefficients are 2.6 × 10^−14^, 2.9 × 10^−11^, 4.8 × 10^−16^, 1.8 × 10^−13^, and 5.2 × 10^−10^ cm^2^ s^−1^ for Fe‐PBA, Mn‐PBA, Co‐PBA, Ni‐PBA, and HEPBA, respectively, leading to a higher rate capability for HE‐PBA. Moreover, the formation energy (E_f_) of HEPBA is more negative compared to all M‐PBAs, indicating its priority in formation over individual M‐PBAs as well as its stability during the process of aqueous precipitation. The enhanced structure stability will lead to reduced dissolutions in aqueous electrolyte, whose capacity is 102.4 mAh g^−1^, with retention of 3448 cycles. Moreover, a high‐entropy strategy can change the charge distribution of electrodes as well. For Mn‐based cathode, especially in aqueous electrolyte, it suffers from Jahn‐Teller distortion and dissolution due to the Mn^3+^ formation in the charge process, leading to poor cycling stability. Wang et al. induced manganese charge redistribution by such a high‐entropy charge compensation mechanism.^[^ ^97^ ^]^ As shown by X‐ray photoelectron spectroscopy (XPS), X‐ray absorption near edge structure (XANES), and extended X‐ray absorption fine structure (EXAFS) spectra (Figure 7H,I), the Mn^2+^ ions in the high entropy PBA cathode remain in a partially oxidized state between +2 and +3 upon charging, rather than being fully oxidized to the Mn^3+^ state. The Jahn‐Teller distortion will be effectively suppressed by such a high‐entropy charge compensation mechanism, which will lead a solid‐solution reaction, an insoluble structure and fast diffusion of K‐ion in electrodes.

The high entropy electrode has also been effectively utilized in the potassium anodes. Tuan synthesized a NaCl‐type high‐entropy metal chalcogenide (HEMC) AgSnSbSe_1.5_Te_1.5_ for potassium anodes.^[^ ^98^ ^]^ Through the conversion reaction, the participation of both inactive and active metals is reconciled in order to generate distinct functional metal nanoparticles and diverse heterointerfaces. The formed heterointerfaces lowered the K^+^ diffusion energy barrier, and the inactive Ag with suitable adsorption energy suppressed the shuttle effect. A similar strategy was also applied to prepare high‐entropy metal disulfide (HES2) colloid clusters for potassium anode materials.^[^ ^99^ ^]^ The clusters with an interconnected network are suitable for K^+^ (de)intercalation and transport. The in situ growth of crystalline‐amorphous heterointerfaces significantly enhances electrochemical kinetics, driven by the abundant grain boundaries that persistently expose active sites. The formed high‐entropy alloys during the conversion reaction show a strong anchoring effect for polysulfides. Finally, such anodes exhibit excellent cycling stability after 1800 cycles (348 mA h g^−1^ at 500 mA g^−1^). The same author also investigated the potassium conversion anode 2D metal phosphorus trichalcogenides by increasing configurational entropy, and prepared high‐entropy CoVMnFeZnPS3 (HEPS3) with a thickness ranging from 6 to 10 nm.^[^ ^100^ ^]^ The binding energy to K^+^ for the high‐entropy anode is the weakest, leading to the lowest diffusion energy barrier of K^+^. In situ‐assembled alloyed structures demonstrate catalytic activity, which suppresses the polysulfide shuttle effect through tailored adsorption energetics while accelerating their redox conversion. Moreover, the “lattice distortion effect” in such high‐entropy anodes effectively disperses the internal stress within the electrode during the process of K^+^ (de)intercalation, mitigating electrode pulverization. The system displays a capacity of 524 mAh g^−1^, high rate with 10 A g^−1^, and high stability for 1000 cycles.

Through the above discussion on various high‐entropy potassium electrode materials, it can be concluded that high‐entropy electrodes play a distinctive role in modulating the electro–chemo–mechanical coupling behavior and improving transport properties of potassium electrodes. In terms of electro–chemo–mechanical coupling characteristics, high‐entropy electrodes exhibit significant differences compared to traditional potassium electrodes in the following aspects: 1) Regulation of phase transformation and stress/strain. For instance, conventional manganese‐based oxides (e.g., KMO) undergo multiple phase transitions (P3‐O3‐P″) during charge‐discharge cycles, where the O3‐P″ transition at high voltage is accompanied by abrupt changes in interlayer spacing, leading to substantial volume expansion and stress accumulation. In contrast, high‐entropy electrodes (HE‐KMO) suppress the detrimental O3‐P3″ phase transformation through high configurational entropy (1.61 R) effect, undergoing only a single P3‐O3 transition with a minimal volume change of 0.31%, thereby significantly mitigating structural stress.^[^ ^101^ ^]^ 2) Preservation of mechanical integrity. Traditional KMO tends to develop visible microcracks on particle surfaces due to stress concentration after cycling, which may eventually lead to electrode pulverization. On the other hand, HE‐KMO, benefiting from the synergistic effects of multiple doping elements, maintains structural integrity without crack formation even after 50 cycles, demonstrating superior resistance to mechanical degradation.^[^ ^101^ ^]^ Indeed, similar beneficial effects of high‐entropy design have also been observed in various potassium electrodes with different reaction mechanisms.^[^ ^102^, ^103^ ^]^

High‐entropy electrodes synergistically enhance transport properties through multiple mechanisms. 1) Improved electronic conductivity: The high‐entropy design enables synergistic regulation of the electronic structure via multielement doping. For example, as for KMF_3_ potassium electrodes, DFT calculations reveal that the band gap of HE‐KMF_3_ is only 0.26 eV, significantly lower than that of conventional KFeF_3_ (1.48 eV), indicating enhanced electronic conductivity.^[^ ^102^ ^]^ Direct current impedance measurements further confirm this improvement, showing that the electronic conductivity of HE‐KMF_3_ (1.38 × 10^−8^ S·cm^−1^) is much higher than that of KFeF_3_ (1.95 × 10^−10^ S·cm^−1^).^[^ ^102^ ^]^ Similarly, in the Cu_3_SbS_4_ potassium anode, the high‐entropy strategy increases the electronic conductivity from 6.4 × 10^−3^ to 30 × 10^−3^ S·cm^−1^.^[^ ^103^ ^]^ 2) Enhanced K^+^ diffusion kinetics: In the case of the KMO potassium cathode, high‐entropy doping with elements such as Mg^2+^ and Al^3+^ expands the interlayer spacing (*c*‐axis parameter increases from 19.091 to 19.225 Å), offering more accessible diffusion pathways for K^+^ ions.^[^ ^101^ ^]^ Consequently, the average K^+^ diffusion coefficient of HE‐KMO reaches 1.04 × 10^−10^ cm^2^·s^−1^ during charging and 1.26 × 10^−10^ cm^2^·s^−1^ during discharging, which is more than three times higher than that of conventional KMO.^[^ ^101^ ^]^ 3) Reduced charge transfer resistance: Using KMO as an example, the high‐entropy design mitigates structural distortion caused by Mn^3+^ ions, leading to a significantly lower interfacial charge transfer resistance (R_CT_) in HE‐KMO compared to its traditional counterpart.^[^ ^101^ ^]^

### 4.4 Measurement and Prediction of Transport Properties

It is crucial to comprehend the transport characteristics for the modification of potassium electrodes. The key to developing high‐performance potassium electrode materials also lies in accurately measuring and even predicting their transport properties. Currently, commonly employed methods for measurement and prediction of transport characteristics include electrochemical testing technology, DFT calculation, alternating current (AC) impedance spectroscopy‐direct current (DC) polarization technique combination (AC‐DC combination), defect chemistry model, etc. In the future, machine learning will emerge as a key approach for transport characteristics investigation.

#### 4.4.1 Electrochemical Testing Techniques

Electrochemical testing technique is the most crucial approach for investigating the transport characteristics of electrode materials. Concerning potassium ion batteries, various types of electrodes such as layered oxides, Prussian blue materials, and polyanionic electrodes have been studied using electrochemical testing techniques to investigate their transport properties.^[^ ^104^ ^]^ Currently, the most common transport characteristic parameter is the diffusion coefficient, which can be measured by a variety of electrochemical testing methods, including CV, electrochemical impedance spectroscopy (EIS), potentiostatic intermittent titration technique (PITT), GITT, etc.^[^ ^105^ ^]^ However, it should be noted that diffusivity values obtained from different methods may vary significantly, thus considering the specific application scope of each method becomes essential. The effective diffusion coefficient throughout the entire redox process can be determined by combining CV with Randles‐Sevcik equation which also allows us to differentiate between diffusion and surface processes based on mathematical relationships between current density and sweep speed. Additionally, EIS, PITT, GITT, and other methods can measure the diffusion coefficient under different potassium contents. The similarity of these methods is that by applying an electrical signal, by tracking the subsequent response, and solving the mathematical equation, the diffusion coefficients at different potassium contents or different potentials are obtained.

**Figure 8:**
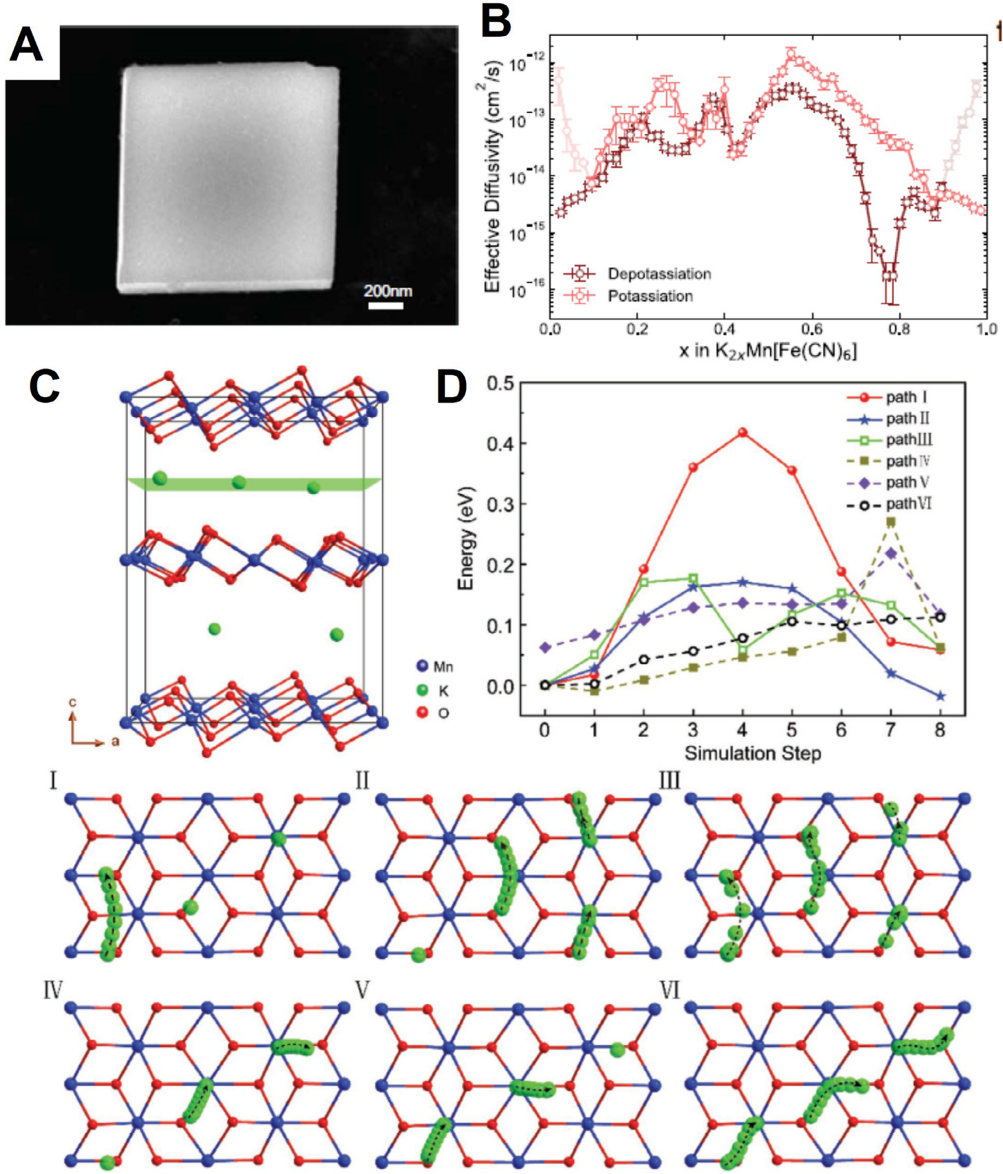
A) SEM of KMF particle. B) Effective diffusivity over composition from Kang‐Chueh GITT. Reproduced with permission.^[^ ^106^ ^]^ Copyright 2024, Springer Nature. C) Schematic illustration K^+^ ion migration and 6 trajectories for K^+^ ion migration. D) Calculated K^+^ migration energy barriers, hopping in P2‐K_0.3_MnO_2_. Reproduced with permission.^[^ ^110^ ^]^ Copyright 2019, Wiley‐VCH.

As mentioned above, when using the GITT method to calculate the diffusion coefficient, we will find that even for the same material, its diffusion coefficient may be different by several orders of magnitude in different reports. This inaccuracy could be due to a variety of reasons, such as unsuitable experimental conditions or uncertainties of corresponding parameters. The difference between the ideal electrode and the real electrode is also one of the important reasons. In principle, the electrochemical technique to determine the diffusion coefficient is based on single‐phase, dense, planar bulk electrodes. However, in the real cases, the electrodes are multiparticle, multiphase, and porous, which leads to significant differences. When GITT is applied to measure the diffusion coefficient for porous electrodes, one of the key factors is the electrochemically active surface area S, which is with an inverse‐square relation. As a result, the uncertainty of S will lead to various D values in orders of magnitude difference. In order to achieve the accuracy of D values by GITT, Pasta et al. prepared K_2_Mn[Fe(CN)_6_] (KMF) with greater morphological homogeneity (**Figure** **8**A).^[^ ^106^ ^]^ To increase the accuracy of S for the determination of D by using Kang‐Chueh GITT technique, homogeneous and monodisperse KMF are obtained. The uniform KMF without agglomeration increases accuracy for the S determination, which is previous a non‐negligible source of error in the determination of D. The uniform 3D framework structure of KMF enabling K^+^ intercalation from all exposed facets, and the geometric surface area of the KMF particle serves as a reliable indicator for S. Since the capacity of 141 mAhg^−1^ is close to the theoretical value (155 mAhg^−1^), indicating the feasibility of estimation of S. Finally, a median D of KMF from (de)potassiation of 5.50 × 10^−14^ cm^2^ s^−1^ is obtained (Figure 8B), which is remarkable lower than previous mischaracterization of D for Prussian blue analog with the D values of 10^−8^–10^−11^ cm^2^ s^−1^, demonstrating the slower transport of K^+^ in the PBA electrodes.

#### 4.4.2 DFT Calculations

In addition to electrochemical testing methods, the transport properties of numerous materials, particularly new ones, can be predicted through DFT calculations.^[^ ^107^, ^108^, ^109^ ^]^ DFT serves as an effective approach for investigating transport properties by simulating diverse pathways for potassium ion diffusion within crystals and determining the activation energy associated with each pathway. This enables identification of the most probable transport routes and provides a detailed atomic‐level description of these pathways.

Yang et al. investigated the K diffusion behavior in P2‐K_0.3_MnO_2_, a K‐Birnessite electrode, by using DFT calculation.^[^ ^110^ ^]^ The K^+^ ion diffusion in P2‐K_0.3_MnO2 was calculated by using CI‐NEB method. In addition to the conventional interstitial ionic and vacancy‐assisted diffusion behaviors, the migration of K^+^ in P2‐K_0.3_MnO_2,_ involving concerted K^+^ ionic diffusion, was also investigated. As shown in Figure 8C,D, there are six K^+^ diffusion trajectories for the simulations. Path I: single K⁺ migrates along the b‐axis; Paths II and III: concerted movement of two (II) or three (III) K⁺ ions along the *b*‐axis; Paths IV & V: two‐K⁺ concerted migration along the *a*‐axis (IV), with Path V further refining this mechanism; Path VI: three‐K⁺ cooperative migration along the *a*‐axis. The calculated activation energy (E_act_) of path I, which is with vacancy‐assisting K^+^ diffusion, is ≈0.42 eV. The value for path I is obviously higher than path II (≈0.17 eV) and path III (≈0.18 eV) with multiple K^+^ ions concerted migration. The E_act_ of path VI (0.12 eV) with three K^+^ ion migrations is even lower than that of paths IV and V (0.27 and 0.22 eV) with two K^+^ ion migrations. Finally, the diffusion coefficient in P2‐K_0.3_MnO_2_ is 1.06 × 10^−8^ cm^2^s^−1^ along path VI, which is much higher than along path IV (3.16 × 10^−11^ cm^2^s^−1^) and path V(2.20 × 10^−10^ cm^2^s^−1^). Therefore, by DFT calculations, it confirms that the concerted K^+^ ions diffusion exhibits a lower activation energy compared with the single K^+^ ion transport, demonstrating the positive interaction of K^+^ with each other concerning the migration barrier decrease. As a result, the fast ionic diffusion kinetics can be obtained by such cooperation of many ions.

In addition, the combination of DFT simulation calculation and experimental methods can be employed for the development and screening of novel potassium electrode materials. Ceder et al. investigated ten potassium polyanionic electrodes (theoretical capacity > 100 mA h g^−1^) based on the Crystal Structure Database for potential cathodes. Among them, electrodes with average voltage smaller than 4.5 V, i.e., K_2_MnP_2_O_7_, K_2_Mn_2_P_2_O_7_F_2_, K_2_Fe_2_P_2_O_7_F_2_, and K_6_V_2_(PO_4_)_4_ were prepared and tested. Due to the high K^+^ migration barrier in the electrodes, as calculated, non‐reversible capacities of such compounds are observed. Finally, K_3_V_2_Cr(PO_4_)_4_ was selected, which was also confirmed experimentally with a high reversible capacity at a remarkably high potential.^[^ ^111^ ^]^

#### 4.4.3 AC‐DC Combination and Defect Chemistry Model Building

**Figure 9:**
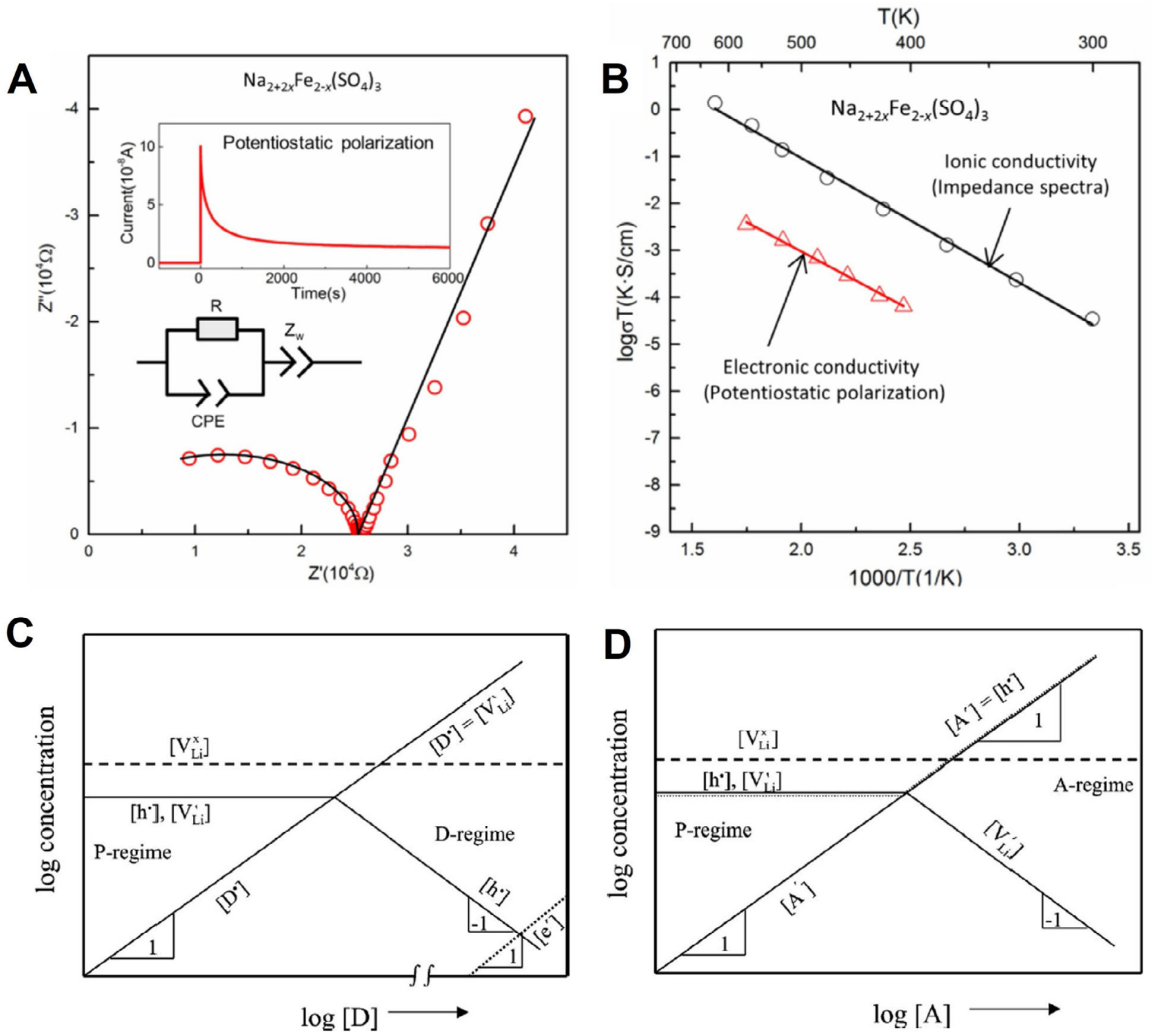
A) AC‐DC measurement and equivalent circuit of NFS. B) Temperature‐dependent ionic and electronic conductivities of NFS. Reproduced with permission.^[^ ^120^ ^]^ Copyright 2016, Wiley‐VCH. C) Defect chemistry models for donor‐doped LiFePO_4_ and acceptor‐doped LiFePO_4_. Reproduced with permission.^[^ ^45^ ^]^ Copyright 2008, IOP.

By measuring the diffusion coefficient, the transport characteristics of potassium ions in the electrode can be understood. However, it is often preferable to determine the intrinsic properties of ionic conductivity and electronic conductivity for subsequent design considerations of the electrode materials. From a solid‐state ionic perspective, we can synthesize carbon‐free single‐phase potassium electrodes. Subsequently, we can measure the ionic conductivity and electronic conductivity at different temperatures separately by assembling an ion‐blocking cell and an electron‐blocking cell, combining AC impedance and DC polarization technology, in order to add more information on transport properties to the database. Similar measurements have been conducted on classical lithium and sodium electrode materials such as LiFePO_4_ (LFP),^[^ ^112^, ^113^, ^114^, ^115^, ^116^, ^117^, ^118^ ^]^ Na_3_V_2_(PO_4_)_2_F_3_ (NVPF),^[^ ^119^ ^]^ Na_2.5_Fe_1.75_(SO_4_)_3_(NFS)^[^ ^120^ ^]^ to deepen our understanding of their transport characteristics. For instance, Yamada's research group studied NFS materials using such methods and obtained measured values of 10^−7^ S cm^−1^ for ionic conductivity and 2 × 10^−9^ S cm^−1^ for electronic conductivity, respectively, thus indicating that the ionic conductivity was two orders of magnitude higher than the electronic conductivity (**Figure** **9**A,B).^[^ ^120^ ^]^ Consequently, subsequent material modifications should focus on improving electronic conductivity. However, unfortunately, because the K‐ion batteries development is still in the early stage, there is still a lack of ionic and electronic conductivity measurements of typical potassium electrode materials by using this method.

Based on the measurements of intrinsic ionic conductivity and electronic conductivity, the defect chemistry models of key materials can be established, which constitute an integral part of predicting material transport properties. Among them, the establishment of a defect chemistry model of LFP is one of the most successful examples.^[^ ^45^, ^112^, ^121^ ^]^ As depicted in the Figure 9C, by measuring various transport properties in different situations, such as donor and acceptor doping, and combining them with fundamental principles of defect chemistry, we have developed a comprehensive defect chemistry model for LFP‐FP that enables prediction of LFP transport characteristics under various doping conditions and lithium activities, thereby facilitating rational material design. However, there are still significant deficiencies in potassium electrode materials in this regard; hence, it is imperative to investigate classical potassium electrode materials, establish corresponding defect chemistry models,^[^ ^122^ ^]^ and summarize as well as predict the transport characteristics of potassium electrodes.

#### 4.4.4 Machine‐Learning

As mentioned earlier, the transport properties of potassium electrodes can be predicted by establishing a defect chemistry model. However, this approach requires a substantial number of doping experiments and measurements of transport characteristics. Unfortunately, there is still a lack of fundamental data for potassium electrode materials in this regard. Machine learning (ML), owing to its capability to address complex tasks and process large‐scale data, is facilitating a transformative shift for electrode materials and systems development.^[^ ^123^, ^124^, ^125^, ^126^ ^]^ ML algorithms can accelerate research applications such as electrode material screening and performance forecasting, electrolyte design, material performance characterization, and manufacturing parameter optimization.^[^ ^127^ ^]^ Therefore, machine learning offers an efficient research method for predicting the transport properties of potassium electrodes.

The field of machine learning (ML) enables the discovery and extraction of patterns from existing data, facilitating the establishment of mappings between input and output data for accurate predictions on new data. Typically, machine learning includes four key steps: 1) Data acquisition involving experiments, computational simulations, or direct collection from databases, including structural information, chemical composition, and material properties, represented by descriptors, in order to construct a comprehensive material library; 2) Feature transformation or selection, i.e., transformation of the material library through feature selection or transformation techniques into samples suitable for enhancing the prediction accuracy of machine learning models; 3) Selection of appropriate machine learning algorithms; 4) Application of machine learning models in material development and performance prediction.^[^ ^127^ ^]^

The application of machine learning methods in predicting transport properties has proven successful for both Li‐ion and Na‐ion batteries. In order to investigate the transport properties of lithium ions, Talei et al. developed a machine learning model that extends the previously proposed optical method for investigating Li^+^ transport with operando microscopy.^[^ ^128^ ^]^ They demonstrated this method by examining the LiMn_2_O_4_ lithiation process as an example, effectively replacing traditional techniques such as GITT, PITT, CV, EIS, etc. This model is capable of measuring diffusion coefficients related to concentration and can be applied to other battery material that exhibits a significant optical response to lithium. Couto et al., on the other hand, proposed a combination of K‐Means machine learning and electrochemical modeling to determine ionic diffusivity and transport properties of sodium electrodes.^[^ ^129^ ^]^ For phase‐change electrodes, they employed K‐means machine learning to screen GITT steps and performed numerical simulations using a pseudo‐2D (P2D) electrochemical model supported by a physically informed algorithm in order to determine their solid‐state diffusion coefficients. Through this approach, they successfully determined that the NVPF electrode's sodium ion diffusion coefficients varied between 9 × 10^−18^ and 6.8 × 10^−16^ m^2^ s^−1^ across 47 steps of GITT characterization.

**Figure 10:**
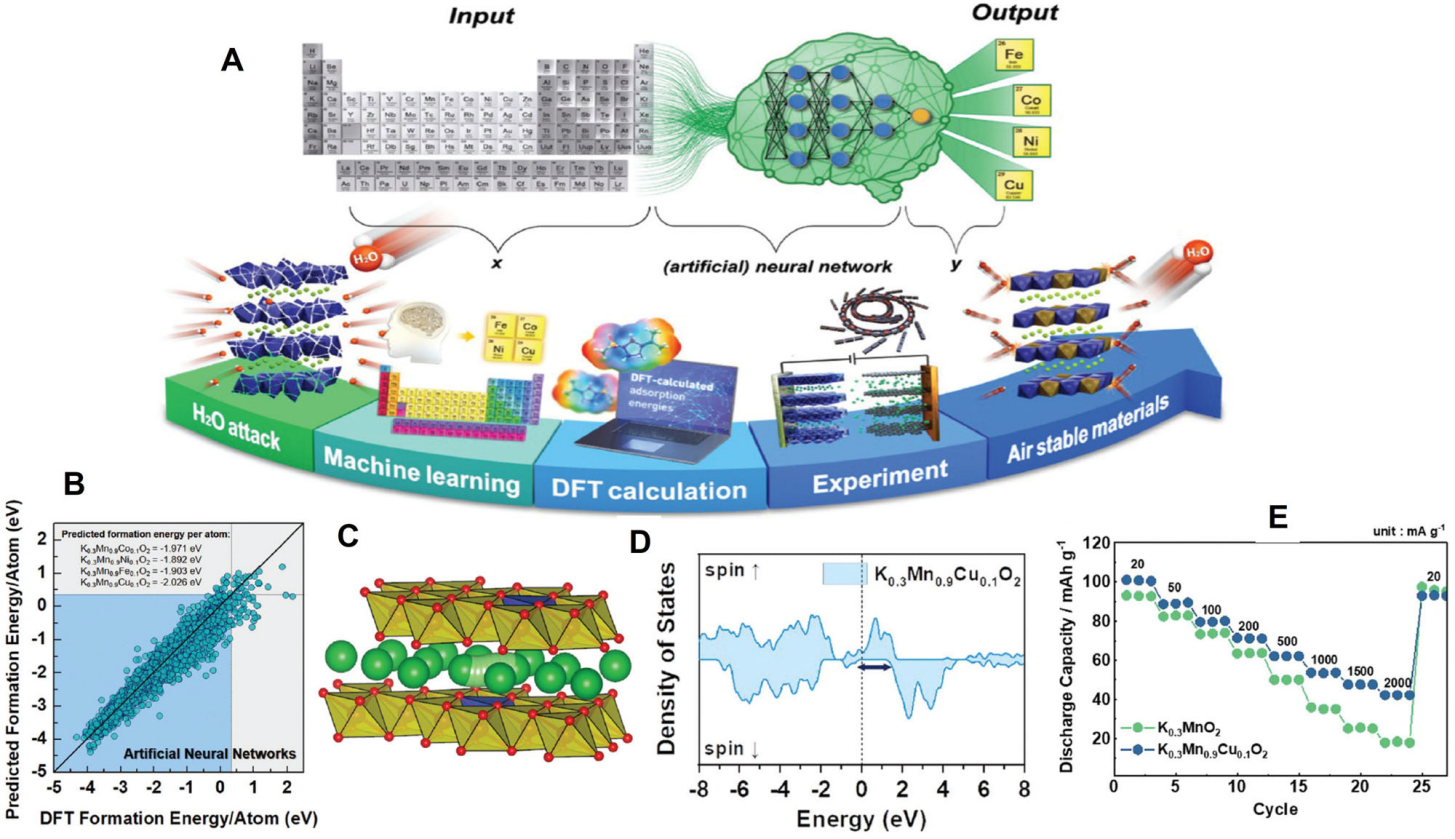
A) Machine learning‐DFT calculations‐ new materials discovery and design experimentally. B) Predicted safety and formation energy per atom by the ML models compared with calculated formation energy per atom. C) Crystal structure and K^+^ diffusion path of P’3‐KMCO visualized using the VESTA program. D) Calculated total DOS of KMCO. E) Rate capability of the KMO and KMCO. Reproduced with permission.^[^ ^130^ ^]^ Copyright 2021, RSC.

The efficient screening of potassium electrodes can be facilitated by machine learning as well. To address the challenges about the structural stability and air stability of layered oxide cathode K_x_MnO_2_, Kim et al. proposed a development platform for potassium layered oxide cathodes based on a combination of machine learning predictions, DFT calculations, and experimental verification for material screening purposes (**Figure** **10**A).^[^ ^130^ ^]^ Initially, a machine learning model was constructed using 27578 entries to predict the stability of crystals and the environmental stability of seven different metal‐doped K_x_MnO_2_. Subsequently, these predicted results were validated through DFT calculations (Figure 10B). It was predicted that P'3 type K_0.3_Mn_0.9_Cu_0.1_O_2_ (KMCO) exhibits favorable air stability and structural stability while also inhibiting charge order and reducing electron localization effects. Additionally, the transport properties of K^+^ were verified and calculated accordingly. Figure 10C,D illustrates the pathway for K^+^ transport, which further confirms that copper doping enhances electronic conductivity and performance. Following this analysis, experimental synthesis was conducted to produce the desired material wherein Cu^2+^ successfully replaced Mn sites, which can inhibit Jahn‐Teller distortion and enhance water sensitivity, improve the crystal structure stability, and ultimately improve the performance. Consequently, even after exposure to ambient air conditions over a 4‐week period, KMCO cathodes demonstrated higher rate capability (Figure 10E) while exhibiting long‐term cycle stability exceeding 100 cycles at 500 mA g^−1^.

Specific capacity is a crucial performance indicator for evaluating battery performance. In the case of intercalation potassium electrodes, Pathak et al. utilized machine learning based on composition features to predict specific capacity.^[^ ^131^ ^]^ Various machine learning models were employed, and the Kernel Ridge regression model was found to be the most reliable using the mean absolute percentage error as an evaluation criterion. The capacity values obtained can determine the number of K ions inserted into each electrode material and verify intercalation stability in the electrode material through DFT calculations.

The application of machine learning for the potassium anodes development is noteworthy as well. For instance, Shen et al. employed machine learning to predict the storage performance of potassium based on layered carbon anodes’ key structural parameters.^[^ ^132^ ^]^ Additionally, Zhou et al. fabricated a layered porous spongy carbon as a potassium anode with notable features such as high sulfur and nitrogen content, abundant defects, and large layer spacing.^[^ ^133^ ^]^ Machine learning was then utilized to establish the correlation between various parameters and performance, further confirming its excellent capabilities. DFT calculations indicate that N/S doping and vacancy defects effectively enhance K^+^ adsorption and storage.

## 5 Size Effects in Potassium Electrodes

Size effects, as the most crucial extrinsic control approach for transport properties in potassium electrodes, are significant in enhancing the K‐ion battery's performance. Notably, the size effect not only significantly enhances transport kinetics but also influences the thermodynamic properties^[^ ^134^ ^]^ of materials and induces changes in potassium storage and transport mechanisms. At the same time, application of the size effect serves as a critical approach to enhance the electro–chemo–mechanical coupling properties of potassium electrode materials. A profound comprehension of size effects is essential for rational design and modification of potassium electrode materials.

### 5.1 Transport Kinetics Enhancement

**Figure 11:**
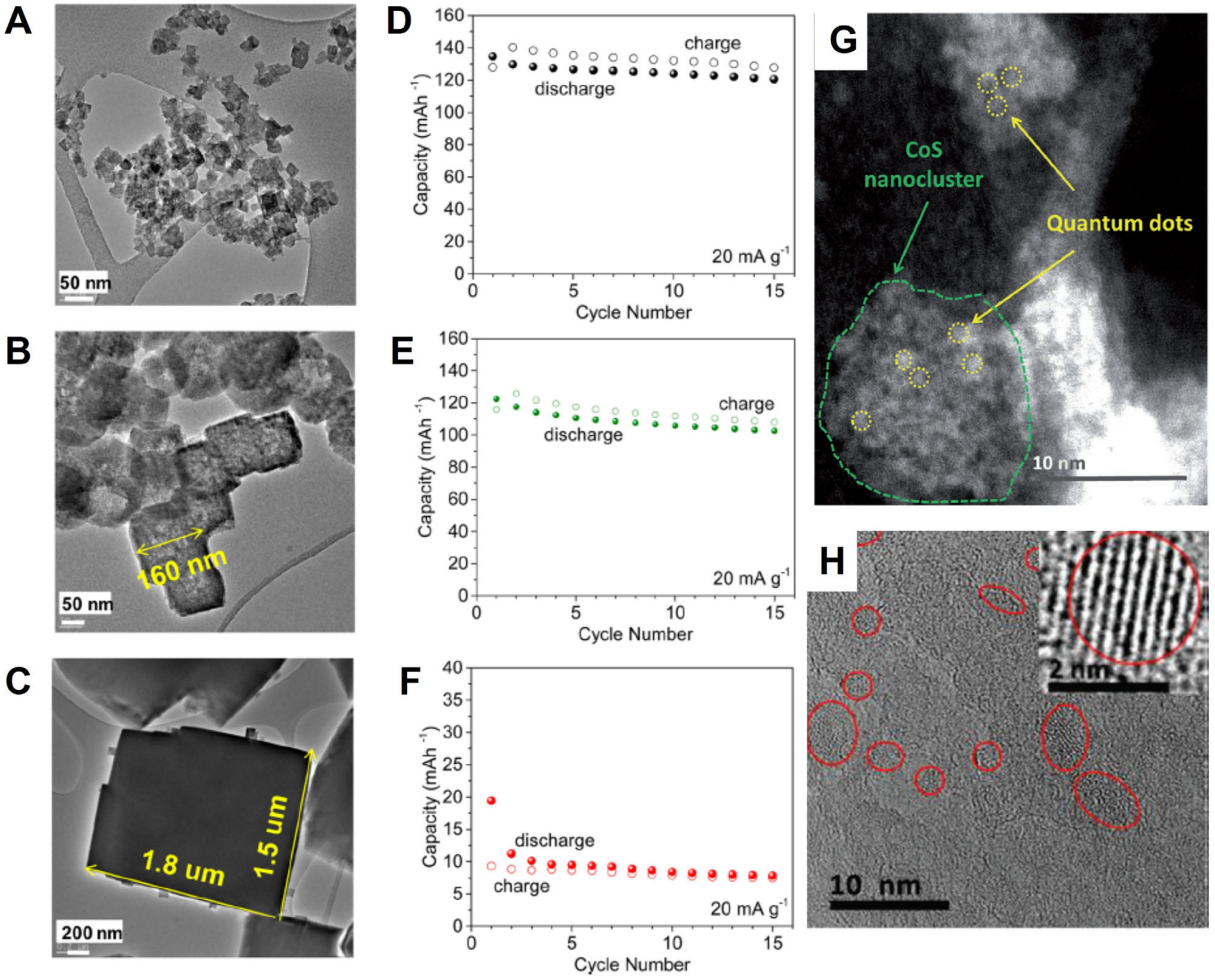
A–C) TEM images and D–F) (dis)charge profiles for KFeHCF with different sizes. Reproduced with permission.^[^ ^145^ ^]^ Copyright 2017, ACS. G) Dark field images for CoS@G‐25. Reproduced with permission.^[^ ^149^ ^]^ Copyright 2017, Wiley‐VCH. H) HRTEM images of the CDs@rGO paper. Reproduced with permission.^[^ ^150^ ^]^ Copyright 2020, Wiley‐VCH.

The diffusion time has a quadratic relationship with the size, as indicated by the formula L^2^/D. Therefore, reducing the size will have an immediate impact on the kinetics enhancement. Irrespective of the morphology of potassium electrode materials (e.g., nanodots, nanowires, nanosheets, or the composite structure between them), shortening the transport distance can increase ion‐electron transport and effectively improve both reversible capacity and rate performance of potassium electrode materials.^[^ ^135^, ^136^, ^137^, ^138^, ^139^, ^140^, ^141^, ^142^, ^143^, ^144^ ^]^ Nazar et al. investigated the size effects of Prussian white cathodes for K‐ion storage.^[^ ^145^ ^]^ By controlling the crystal sizes of K_1.7_Fe[Fe(CN)_6_]_0.9_ in the range of micron, submicron, or nano crystallites through a solution chemistry method, an obvious effect of size on performance was observed (**Figure** **11**A–C). The optimal cathodes with 20 nm crystallites exhibit a 140 mAh g^−1^ capacity, whose value is almost the theoretical capacity during the discharge process, while for the electrode with the crystalline size ≈160–200 nm, the capacity is 125 mAh g^−1^. Nonetheless, for micron crystals, only a very limited capacity of 10 mAh g^−1^ is observed (Figure 11D–F). Additionally, for nanosized potassium cathodes, the energy density is ≈500 Wh kg^−1^, which is comparable to the optimal Na counterpart cathodes.

Similar size‐dependent capacity behaviors are also found in hematite potassium anodes (α‐Fe_2_O_3_ nanoparticles).^[^ ^146^ ^]^An unusual capacity increase was highly related to the particle size. As the sizes decreased from 115 to 20, 5, and 2 nm, the capacities increased from 312 to 360, 874, and 1000 mAh g^−1^ at 0.1 A g^−1^. Nonetheless, in this case, the partial increase in capacity is from the electrolyte decomposition due to the smaller particle sizes. Finally, the optimized size of the 5 nm‐sized hematite particles displayed the best capacity retention after 600 cycles. It highlights the importance of considering the adverse impacts of small nanoparticles as well.

In addition to nanoparticles, the preparation of 1D nanowires is an effective method to improve the electron/ion transport.^[^ ^147^ ^]^ Lei et al. synthesized highly nitrogen‐doped carbon nanowires as a soft carbon anode.^[^ ^148^ ^]^ This nanowire anode exhibits a cross‐linked morphology, and the hollow interior, whose sidewall is ≈20 nm thick with an 30–40 nm inner diameter. The surface is rough with structural defects, which are favorable for K^+^ storage. The nanofiber electrode exhibits capacities of 248 mAh g^−1^ at 25 mA g^−1^, and the capacity is still 101 mAh g^−1^ at even 20 A g^−1^. The capacity maintains 146 mAh g^−1^ at 2 A g^−1^ even after 4000 cycles.

In order to fully exploit the kinetic advantages of small particles, by continuously reducing particle sizes, nanodots or even quantum dot materials can be obtained. By preventing the agglomeration of quantum dot materials, their combination with graphene enables the formation of 2D or 3D composite structures that possess both kinetic advantages and structural stability. This represents a crucial strategy for advancing potassium electrodes. Guo et al. prepared a cobalt sulfide and graphene (CoS@G) composite for potassium anodes, which is built up from interconnected CoS quantum dot nanoclusters anchored on graphene uniformly.^[^ ^149^ ^]^ In the optimized CoS@G‐25 composite (25%GO), there are CoS nanoclusters (10–20 nm) with a much smaller size composed of CoS quantum dots (<2 nm) than the pristine CoS homogenously adhered to the graphene nanosheets (Figure 11G). Such structure exhibits a highly conductive network, high structural stability, large surface area, leading to excellent electrochemical performance. For instance, it shows a very high capacity of 310 mA h g^−1^ after 100 cycles at 500 mA g^−1^. Furthermore, its rate performance is even better than its sodium counterpart. Lu et al. proposed a freestanding 3D hybrid architecture, i.e., carbon dots dispersed on graphene (CDs@rGO), for potassium anodes.^[^ ^150^ ^]^ The graphene sheets are stacked and interconnected to form a well‐ordered multilayer structure. Numerous cavities of varying sizes are present within this structure, providing enough space for electrolyte, whose 3D interconnected channels facilitate electrolyte penetration and K^+^ diffusion. Furthermore, the vertical and horizontal connections between graphene sheets contribute to the high electronic conductivity, enabling rapid electron conduction in both directions. The size distribution of carbon dots is ≈2 ± 0.4 nm (Figure 11H). At 100 mA g^−1^, such anode exhibits the high reversible capacity of 310 mAh g^−1^. It maintains at 244 mAh g^−1^ (at 200 mA g^−1^) even for 840 cycles. The improved transport behaviors of K^+^ and electrons within the anode result in high‐rate capability, whose capacity is still 185 mAh g^−1^ even at 500 mA g^−1^.

### 5.2 Thermodynamic/Intrinsic Properties Regulation

Reducing the electrode size, besides the obvious enhancement on the transport properties, can also effectively regulate the thermodynamic behavior of the material and the intrinsic characteristics,^[^ ^151^, ^152^ ^]^ and the change of such intrinsic characteristics will in turn modulate the transport properties. In addition, for some electrode materials, in the case of micron or larger size, there is no electrochemical activity; only when the size is small enough can they show considerable electrochemical performance, such as NaFePO_4_. Therefore, by reducing the material's size to regulate the material's intrinsic properties, it is also possible to screen new electrode materials.

#### 5.2.1 The Equilibrium Potential of Electrochemical Reactions

When an electrochemical reaction occurs, it corresponds to a thermodynamic equilibrium potential. Whether the electrode material undergoes an intercalation reaction or a conversion reaction, it will correspond to a thermodynamic equilibrium potential. This potential is one of the electrode's intrinsic properties. For electrode materials, the equilibrium potential is closely related to their electrochemical behaviors, and regulating and understanding their changes are crucial for thermodynamic control. The size effect of the equilibrium potential is not limited to the electrode materials of potassium ion batteries, but is an intrinsic characteristic of the electrode materials.

For instance, we have investigated equilibrium potential variations of LiFePO_4_ in terms of sizes and crystallinity.^[^ ^43^ ^]^ The equilibrium voltage of the electrode material is determined by the chemical potential of lithium (Equation 1). When the size of the electrode material changes from microns to nanometers scale, its chemical potential will correspondingly vary, accompanied by an additional energy correction term that depends on particle size, surface tension, and molar volume (Equation 2). For different reaction mechanisms, such as single‐phase solid solution or two‐phase transformation reactions, the molar volume represents either the partial molar volume of lithium in a phase or the molar volume of a phase itself. In terms of amorphous phases, they can be considered as limiting cases of nanocrystals with atomic‐scale sizes.

(1) $$E=-\frac{1}{F}\left(\mu_{\alpha }-\mu_{\beta }\right)$$

(2) $$\mu_{nano}-\mu_{bulk}=\left(\frac{2\bar{y}}{\bar{r}}\right)v$$

The changes in the equilibrium potential of LiFePO_4_ are attributed to variations in the excess chemical potential of lithium due to the size changes. These variations differ depending on the crystallinity (nanocrystalline and amorphous) and lithium storage mechanisms (i.e., single‐phase solid solution or two‐phase phase transformation). Please refer to Ref. [43] for specific variations in the chemical potential of lithium and detailed derivation processes.^[^ ^43^ ^]^ It is worth noting that these findings not only apply to LiFePO_4_ but also extend to electrodes used in K‐ion batteries, as they adhere to the same principle.

However, owing to the expanded ionic dimension of potassium ions, the equilibrium potential of K^+^ intercalation in the same material differs from that of Li^+^ and Na^+^. For instance, as mentioned in the references,^[^ ^6^ ^]^ the variation in equilibrium voltages of AFePO_4_ and AFeSO_4_F (A = Li, Na, K) frames in the intercalation of different ions is calculated. For the olivine FePO_4_ structure, based on calculations, the K insertion is less favorable, leading to a lower K insertion potential compared to lithium and sodium. While for AFeSO_4_F (A = Li, Na, K) structure, the potential of K insertion (KFeSO_4_F) increases compared to that of lithium and sodium, due to spacious cavities designed to accommodate alkali ions in KFeSO_4_F. The findings suggest that K compounds demonstrate more pronounced structural preferences compared to Na compounds due to their larger sizes. In conclusion, both the size of the electrode material and the size of the intercalated ion significantly influence the equilibrium potential and should be carefully considered.

#### 5.2.2 Enhancement of Thermodynamic Reversibility

With the reduction in particle size, the kinetic characteristics are gradually enhanced, while the polarization is weakened and the reversibility of electrochemical reactions is improved. In principle, when the size falls below a specific threshold, it can approach thermodynamic equilibrium indefinitely and enable complete reversibility of electrochemical processes.

**Figure 12:**
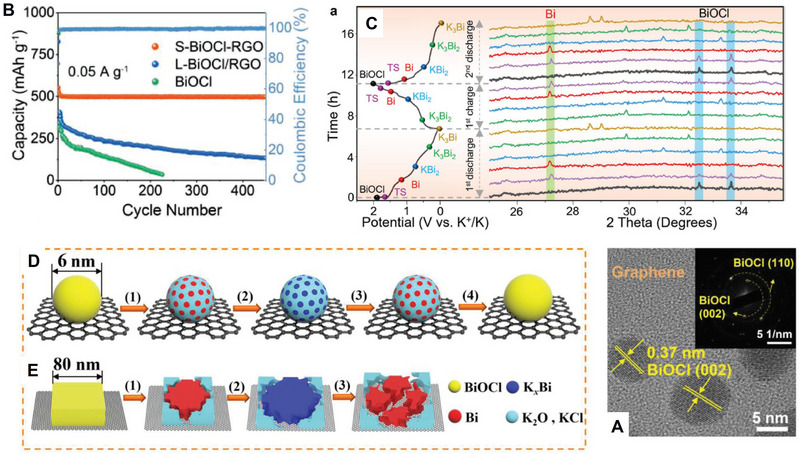
A) HRTEM image and SAED pattern (inset) of S‐BiOCl‐RGO. B) Cycling performance for various BiOCl samples with different sizes. C) In situ XRD for the sample of S‐BiOCl‐RGO. Schematic of structural evolution process during charge/discharge procedure of D) S‐BiOCl‐RGO and E) L‐BiOCl/RGO. Reproduced with permission.^[^ ^153^ ^]^ Copyright 2022, Wiley‐VCH.

BiOCl has been investigated as a potassium anode material owing to its high theoretical capacity. The van der Waals gaps in BiOCl offer fast and favorable ion transport pathways for large K^+^, and formed Bi will increase the electronic conductivity. However, various reported bulk or nanostructured BiOCl still exhibit low capacity and poor cycling performance. Xu et al. prepared several ultrasmall BiOCl samples of≈6 nm (as shown in **Figure** **12**A) with a nanoconfinement strategy, which were uniformly anchored on nanosheets of rGO.^[^ ^153^ ^]^ The size of BiOCl can be controlled: strongly aggregated GO or highly dispersed GO leads to BiOCl with larger sizes; the weakly stacked GO results in ultrasmall BiOCl due to a spatially confined nanoreactor from such stacked GO. Endowed by the ultrasmall size, fully reversible potassium storage is obtained. S‐BiOCl‐RGO displays a record high capacity of 521 mAh g^−1^ at 0.05 A g^−1^. The capacity is still 497 mAh g^−1^ after 450 cycles (94.3% capacity retention), which demonstrates the high cycling reversibility. In contrast, for the sample of BiOCl and L‐BiOCl/RGO, capacities decay very fast (as shown in Figure 12B). As indicated by in situ XRD and schematic of structural evolution (Figure 12C–E), due to the ultrasmall BiOCl, the conversion reaction and alloying reaction can propagate radially and uniformly within such entire small nanoparticles, which can promote the reaction to complete. The formed nanoparticles can keep their structural integrity, discreteness, and ultrasmall size. Ultrasmall products, i.e., K_3_Bi, K_2_O, and KCl co‐penetrated in a highly integrated manner, leading to a fully reversible reaction process (BiOCl → Bi → K3Bi → Bi →BiOCl). However, for larger BiOCl particles (≈80 nm), the conversion and alloying reaction cannot propagate uniformly, resulting in serious structural deformation and phase separation. Aggregation of K_3_Bi is partially encased in a KCl and K_2_O shell. K_3_Bi with a large size leads to a crack. The small interface contact area between K_3_Bi, K_2_O, and KCl results in nonreversible reactions.

#### 5.2.3 Intrinsic Mechanical Properties

With the variation in material size, its intrinsic mechanical properties undergo significant changes.^[^ ^154^ ^]^ Specifically, upon ion intercalation, materials of different sizes exhibit distinct macroscopic behaviors such as expansion and even cracking. For instance, in the case of silicon anodes, the mechanical stress during particle expansion decreases with the reduction of particle size. Several studies have indicated that when the particle size is below 150 nm, pulverization of Si particles will not occur.^[^ ^155^ ^]^

**Figure 13:**
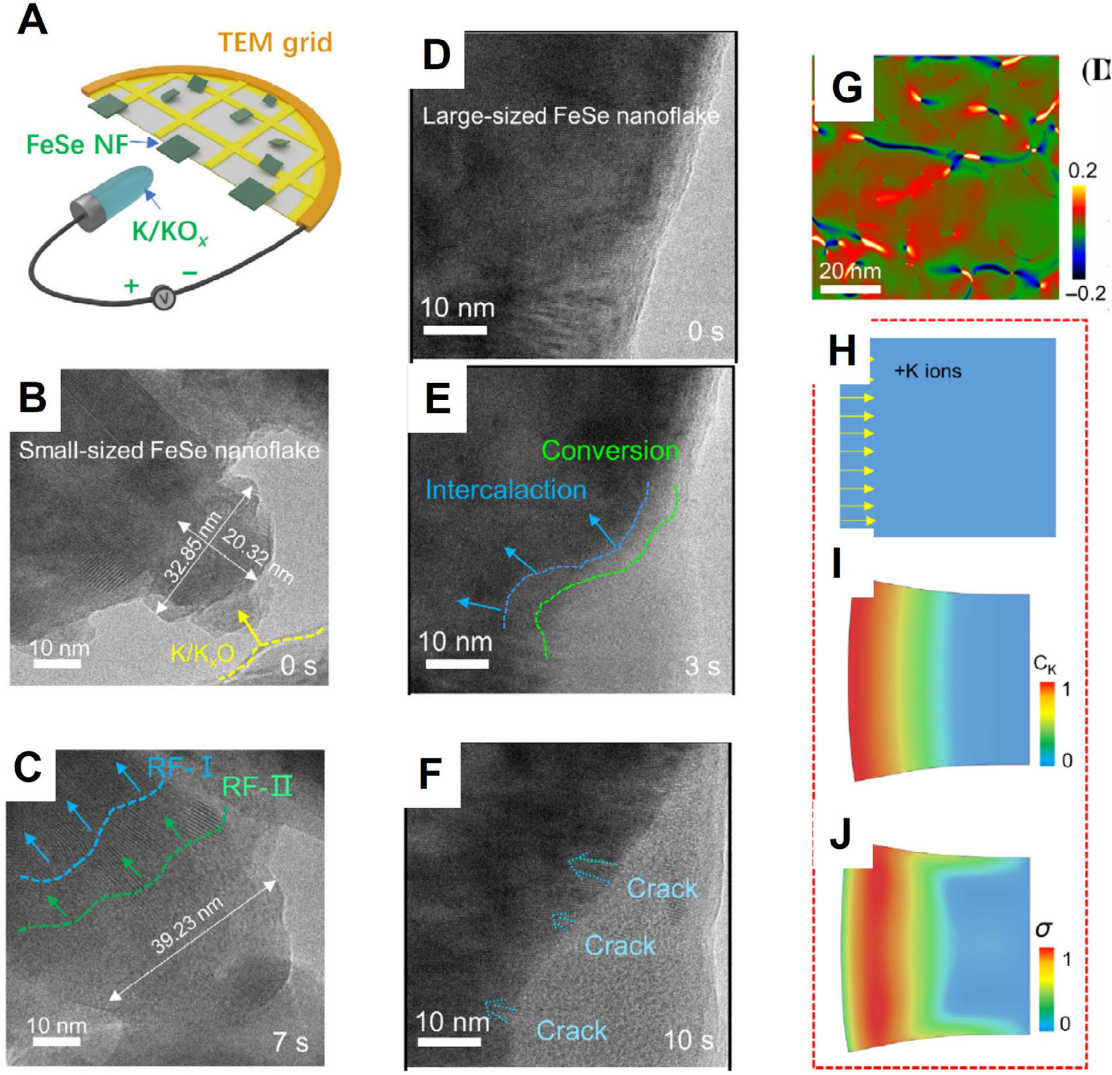
A) Schematic diagram of in situ electrochemical potassium storage. B,C) Time‐sequenced TEM images for FeSe nanoflake with small size at the first potassiation. D–F) Time‐sequenced TEM images for FeSe nanoflake with a large size at the first potassiation. G) Geometric phase analysis (GPA) test. H–J) Finite element analysis (FEA) analysis for electrochemo–mechanical properties. Reproduced with permission.^[^ ^156^ ^]^ Copyright 2023, Wiley‐VCH.

The size effect plays a crucial role in governing the electro–chemo–mechanical coupling behaviors of potassium electrodes. Designing nanostructures, including size reduction and diverse nano‐morphology engineering, represents an effective strategy to mitigate volume deformation and alleviate stress accumulation. Real‐time atomic‐scale investigation of FeSe anodes for potassium storage was made by in situ high‐resolution transmission electron microscopy (HRTEM) (**Figure** **13**A).^[^ ^156^ ^]^ During the introduction of potassium, intercalation and conversion reactions sequentially occur for FeSe. The initial intercalation behavior exhibits size dependence. For small‐sized FeSe, apparent expansion is observed during K^+^ intercalation (Figure 13B,C); while for FeSe with a large size, along the ionic diffusion direction, cracks are formed (as shown in Figure 13D–F). The significant generation of stress and propagation of cracks arise from the coupled impact of electrochemical and mechanical interactions, which is investigated by geometric phase analysis (GPA) and finite‐element analysis (FEA). An insertion interface where the diffusion of potassium ions resulted in progressive development of significant stress, primarily alleviated through the crack initiation (identified by GPA, Figure 13G). The fast propagation of cracks further facilitated the K^+^ diffusion and expedited the intercalation reaction. As shown in FEA (Figure 13H–J), there is a convex geometry for the reaction front (RF), and the stress distribution resulting from the reaction‐induced concentration profiles is also depicted. The RF's convexity‐induced stress fields drive boundary‐layer cracking, concurrently boosting K⁺ diffusion kinetics in reaction progression. Moreover, due to the size‐dependent mechanical properties, FeSe with a small size exhibits better cycling performance compared to that of larger ones, and with better maintained structural integrity.

### 5.3 Size Effects in Transport and Storage Mechanisms of Potassium

In addition to the influence of kinetics and thermodynamics of potassium electrodes, the size effects will also significantly change the transport and storage mechanism of potassium ions, thereby impacting the design strategy for potassium electrode materials.

#### 5.3.1 Interface Effects

As the electrode size decreases gradually, the proportion of the bulk phase and interface changes gradually. The interface will assume a progressively significant role in the storage and transport of potassium ions as the particle size decreases.^[^ ^157^, ^158^, ^159^ ^]^

**Figure 14:**
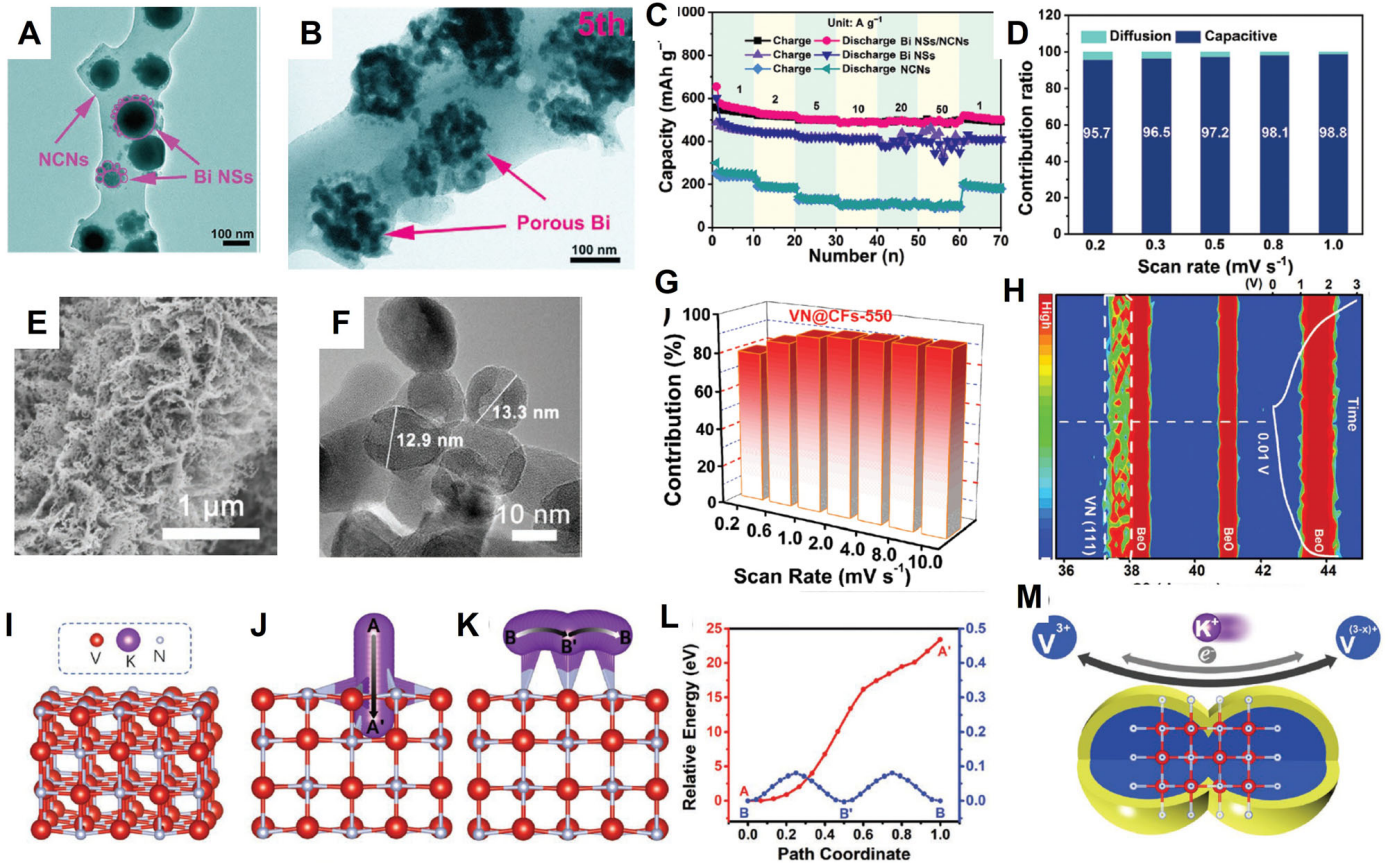
A) TEM images of Bi NSs/NCNs. B) TEM images of Bi NSs/NCNs in the DME‐based electrolyte after cycles. C) Rate performance of Bi NSs/NCNs, Bi NSs, and NCNs. D) Capacitive‐ and diffusion‐controlled capacities contribution. Reproduced with permission.^[^ ^160^ ^]^ Copyright 2021, Wiley‐VCH. E) SEM of VN@CFs‐550. F) HRTEM images for VN@CFs‐550. G) Pseudocapacitive contributions for VN@CFs‐550 at different rates of scan. H) In situ XRD of VN@CFs‐550. I) Optimized VN unit; J) Intercalation in the bulk VN paths of K^+^ (A→A’); K) K^+^ diffusion between the two adjacent adsorption sites (B→B’); L) Relative energies for such two paths; M) The storage mechanism for K^+^. Reproduced with permission.^[^ ^161^ ^]^ Copyright 2022, Wiley‐VCH.

In addition to the size effect of the material itself, certain electrochemical reactions, such as the conversion reaction, can further promote the nanosizing of the electrode material, thus making the interface effect more prominent. Jiang et al. prepared Bi/C composite potassium anodes, i.e., Bi nanospheres embedded in 3D N‐rich carbon nanonetworks (Bi NSs/NCNs).^[^ ^160^ ^]^ As shown by TEM (**Figure** **14**A), Bi with sizes ≈2 to 140 nm is restricted by NCNs. In addition, ball‐cactus‐like Bi NSs are formed, i.e., smaller Bi domains exhibit peripheral arrangement encircling larger Bi cores. Bi NSs/NCNs exhibit excellent rate performance, whose capacities are 565, 524, 501, 491, 490, and 489 mAh g^−1^ at 1, 2, 5, 10, 20, and 50 A g^−1^, respectively (Figure 14C). Such high‐rate capability is quite impressive for potassium anode materials. Its excellent high‐rate performance and fast kinetics can be attributed to the interface effects. After electrochemical cycling of the conversion reaction, the particle size will further decrease, and the surface area of the pore will increase. As shown in Figure 14B, after five cycles, Bi NSs exhibit smaller particle sizes and a porous structure (the diameter of pores≈4–11 nm). The results indicate that the dominant mechanism for capacity is primarily capacitive, with the contribution increasing from 95.7% to 98.8% as rates of scan increase (Figure 14D). This significant contribution from capacitance provides additional capacity and facilitates ultrafast transport kinetics of electrons/ions.

The size of both the electrode material and the intercalation ions may synergistically contribute to the promotion and enhancement of the interface effect, i.e., surface redox pseudocapacitance, which relies on a Faradaic charge‐transfer process occurring predominantly within the surface region or its immediate vicinity of the material. Its kinetic process, controlled by the surface, is much faster than the diffusion‐controlled process in the solid‐state electrode. Moreover, for surface redox pseudo‐capacitive, there is no phase transition, which effectively ensures the structural stability of electrode materials in long cycling. Li et al prepared carbon fiber‐supported nanostructured sheets assembled from VN particles (VN@CFs) to form a free‐standing potassium anode.^[^ ^161^ ^]^ The electronic conductivity of VN is 1.17 × 10^6^ S m^−1^, which is higher compared to graphite. For the lithium storage, it can store the lithium by conversion reaction with a high capacity higher than 800 mAh g^−1^. However, it is hard for the large K^+^ intercalate in the VN lattice owing to the narrow interlayer distances (i.e., VN (002) –0.214 nm). As a result, potassium storage will rather occur on the surface of VN instead of the conversion reaction. As in Figure 14E,F, the VN nanosheets are made up by cross‐linked particles and maintain a flaky and porous structure. Moreover, as displayed in Figure 14G, the surface‐dominated contribution enhances from 78.8% to 93.3% as the sweep rate increases from 0.2 to 10.0 mV s^−1^. In situ XRD was applied for potassium storage mechanism investigation (Figure 14H). The (111) diffraction signals of VN retain their original intensity and positional integrity relative to the untreated reference sample. XRD analysis reveals exclusive presence of VN at full discharge, with no detectable K_3_N, V, or other phases. This absence of potassium ion intercalation or phase conversion phenomena confirms a dominant surface‐mediated pseudocapacitive charge storage mechanism. From a thermochemical point of view, ΔG of reactions between Li^+^ or K^+^ and VN are −11.9 and −2.0 eV, respectively, which demonstrates that both conversion reactions are thermodynamically feasible and spontaneous. However, the conversion reaction between Li^+^ and VN exhibits a higher thermodynamic advantage compared to K^+^. In the real case, the reaction is primarily linked to its kinetics in practice, particularly for K^+^ with a larger size. Based on DFT calculations, a substantial energy barrier of 23.4 eV governs K^+^ migration from surface to subsurface regions along the <002> crystallographic pathway (A→A“), rendering it impractical for K^+^ to be incorporated into the VN lattice. Conversely, optimal positioning of K^+^ occurs atop the nitrogen atom, exhibiting an adsorption energy of −6.78 eV. On the (002) surface of VN, migration along the B→B” path encounters a significantly lower energy barrier value of only 0.08 eV (Figure 14I–M). This exceptionally low diffusion barrier facilitates fast migration of adsorbed K^+^ on the VN surface rather than its insertion into the lattice.

When the composite is prepared, a heterostructure interface will be formed between different materials.^[^ ^162^, ^163^ ^]^ As the material size decreases, the proportion of the heterojunction interface will significantly increase, resulting in a greater influence. Thermodynamically, the presence of a heterojunction interface enhances interfacial potassium storage. From a kinetics perspective, the interface facilitates interfacial potassium ion transport.^[^ ^164^ ^]^ Zhao et al. prepared BiSb nanoconfined in a 3D carbon framework (BiSb@C) for potassium anodes.^[^ ^165^ ^]^ 20–30 nm BiSb nanoparticles are fully coated by graphene‐like thin carbon layers (≈5 nm), which are embedded into 15 nm thin carbon nanosheet. DFT simulations reveal that dual‐metallic interfacial synergy significantly facilitates potassium ion storage kinetics at metal/graphitic heterointerfaces. Adsorption energy of BiSb/graphene interface is lowest (−1.36 eV), compared to those of Sb/graphene interface (−1.24 eV) and Bi/ graphene interface (−1.13 eV), indicating that binary alloy not only can store K at the interface, but also facilitated for the spontaneous K adsorption to efficiently enhance interface storage, owing to the more synergistic effects of BiSb/coating layers. Sun et al. prepared black phosphorus/red phosphorus heterostructure on multiwalled carbon nanotubes (BRPH@MWCNT), which exhibited a high capacity of 923 mAh g^−1^ at 0.05A g^−1^, and high rate capability of 335 mAh g^−1^ even at 1Ag^−1^.^[^ ^166^ ^]^ Abundant heterostructure interfaces are able to effectively improve the adsorption, injection, and immigration of K^+^. The BRPH exhibits a more negative adsorption energy of −0.83 eV compared to that of red P (−0.36 eV), which indicates an enhanced adsorption of K^+^ at the interface of the heterostructure. Calculated the diffusion pathway of K^+^ reveals a much lower energy barrier (0.61 eV) for the black P/red P heterostructure, in contrast to 1.51 eV for red P alone, suggesting improved reaction kinetics facilitated by the heterostructure.

For potassium electrode materials, interface engineering is not only a strategy to modulate their transport characteristics but also an effective approach to address chemical–mechanical challenges. In the case of nanomaterials, the significance and proportion of interfaces increase, thereby making interface regulation even more critical. By optimizing electrolytes and additives, it is possible to control the composition and mechanical robustness of the CEI/SEI layers, suppress transition metal dissolution, and enhance cycling stability. Liu's group proposed a dual‐additive modification strategy with the synergistic effect between KSeCN and LiDFOB. This approach aims to enhance the electrolyte stability and construct a robust CEI on the KFeHCF surface. Specifically, K_2_CO_3_ contributes to improving the viscoelasticity of the CEI, while PEO enhances its mechanical properties and facilitates the construction of high K^+^‐conductive networks. Such CEI effectively mitigates severe side reactions at a 4.5 V high voltage, thereby maintaining the KFeHCF structural integrity and significantly improving its cyclic performance.^[^ ^167^ ^]^

#### 5.3.2 Size Effects of Pores

Porous material is a typical morphology for electrodes, and the performance of these materials is profoundly influenced by different pore sizes and pore structures. For the investigation of K‐ion batteries, the size effect of pores becomes more significant owing to the larger size of K^+^ compared to Li^+^ and Na^+^. Different pore sizes exhibit distinct effects, while the synergistic impact of multiple pores also cooperatively regulates the electrochemical performance of potassium storage.^[^ ^168^, ^169^, ^170^, ^171^, ^172^, ^173^, ^174^, ^175^, ^176^, ^177^ ^]^ In addition, the porous structure can be as a buffer for potassium electrode volume expansion, thereby representing an efficient strategy to improve electro–chemo–mechanical coupling behaviors.

**Figure 15:**
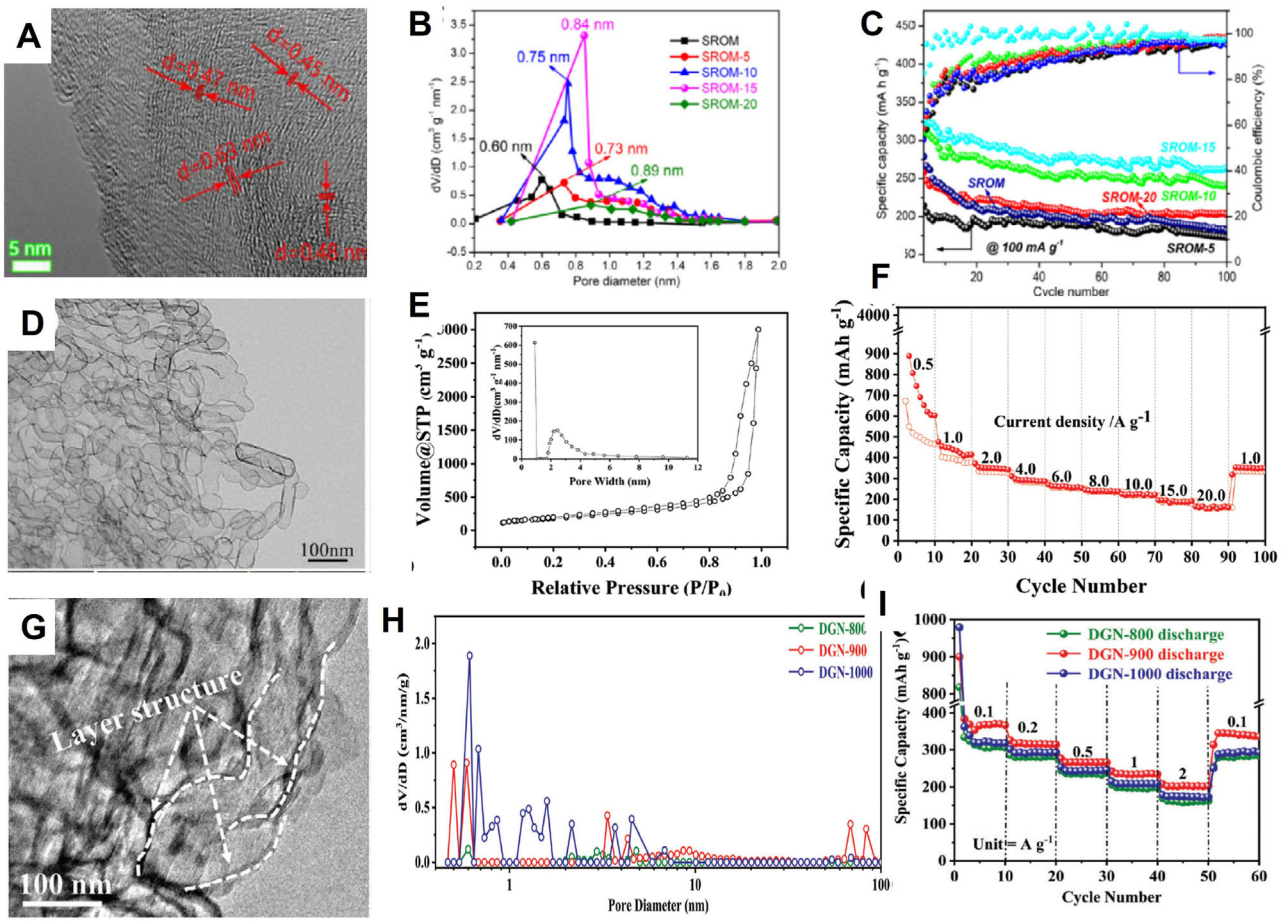
A) HRTEM image of SROM‐15. B) Size distribution of pore in the range of 0–2 nm for five samples in SROM‐15. C) Cycling performance of five samples. Reproduced with permission.^[^ ^178^ ^]^ Copyright 2021, Elsevier. D) TEM images of N‐CNS. E) Pore size distribution and Nitrogen adsorption–desorption isotherm of N‐CNS. F) Rate performance of N‐CNS. Reproduced with permission.^[^ ^179^ ^]^ Copyright 2020, Wiley‐VCH. G) TEM images for DGN‐900. H) Size distribution of pore for DGN‐1000, DGN‐900, and DGN‐800. I) Rate performance of DGN‐1000, DGN‐900, and DGN‐800. Reproduced with permission.^[^ ^169^ ^]^ Copyright 2023, Wiley‐VCH.

Carbon material is the most important anode for potassium storage, and it is significant to control the structure and size of the pores in the material to regulate the storage and transport properties of potassium ions. As for the micropore structure, Kong studied the impact of pore structure on the K‐ion battery performance by synthesizing a series of short‐range order ultra‐microporous carbon (SROM) with varying micropore sizes through a combination of ball milling and pyrolysis.^[^ ^178^ ^]^ They discovered that the pore size significantly influenced the electrochemical performance of the batteries, identifying 0.84 nm as the optimal pore size for potassium ion storage at the micropore scale. As depicted in **Figure** **15**A, SROM‐15 exhibits a well‐developed 3D microporous structure composed of interconnected carbon nanosheets. The nanoporous walls consist of disordered graphite nanocrystals with a layer spacing ≈0.5 nm larger than that of graphite (0.33 nm). N_2_ adsorption–desorption isotherm analysis confirms that SROM‐15 possesses micropores within the range of 0–1 nm diameter. Notably, SROM‐15 shows a specific surface area 721cm^2^ g^−1^ along with a micropore diameter of 0.84 nm (Figure 15B). The sample SROM‐15 shows a capacity of up to 264 mAh g^−1^ after 100 cycles at 100 mA g^−1^ (Figure 15C), and still has a capacity of 149 mAh g^−1^ at 5A g^−1^. This can be attributed to its appropriately sized pores, which provide suitable storage space for effective adsorption and desorption processes involving potassium ions. In contrast, despite having the largest specific surface area among all samples (1283cm^2^g^−1^), SROM‐5 exhibits relatively low capacity (173 mAh g^−1^) due to its excessively small pore size which hinders electrolyte ions from entering into its interior pores effectively utilizing only part of the available specific surface area thereby leading to reduced capacity.

For mesoporous structures, the high reversible capacity as well as long cycling of anode materials for potassium storage were achieved by Yu et al. through the synthesis of N‐doped 3D mesoporous carbon nanosheets (N‐CNS), in combination with morphology, defects, and structural engineering.^[^ ^179^ ^]^ The N‐CNS obtained using this method exhibits an ultrathin nanosheet structure with hierarchical pores, ultrahigh pyridinium/pyrrole nitrogen content, and extended layer spacing. As shown in Figure 15D, the nanosheet is composed of 50–100 nm carbon nanoparticles with a 3D hollow structure that facilitates electrolyte penetration and ion/electron diffusion. Nitrogen adsorption–desorption isotherm analysis reveals a typical type IV isotherm with H1 hysteresis ring, indicating a mesoporous structure (Figure 15E). The pore size of N‐CNS measures ≈2.5 nm, while the specific surface area reaches 654 m^2^g^−1^. It exhibits a capacity of 321mAh g^−1^ for cycling 5000 times at 5A g^−1^ as a potassium anode. High‐rate capability is as follows: 464 and 161 mAh g^−1^ at 0.5 and 20A g^−1^ (Figure 15F). It demonstrates that the mesoporous size provides suitable channels for the K^+^ diffusion in the electrodes, facilitating fast ion transport and improving rate performance as well as reaction kinetics in batteries. Furthermore, the pore size, along with other characteristics such as nitrogen doping and expanded layer spacing, collectively influence electrochemical performance. The appropriate pore size combined with high pyridinium/pyrrole nitrogen doping levels and enlarged layer spacing enables N‐CNS to exhibit outstanding performance for K‐ion batteries.

For the macroporous structure, Wang et al. prepared a super‐structured carbon hexahedron (DG‐900) and investigated the impact of its structural characteristics on potassium ion storage performance.^[^ ^169^ ^]^ The material has the structure of large porous defect graphite nanosheets, which contain large pores ≈83 nm. The volume of large pores is up to 25.2cm^3^g^−1^, and the large pores are interconnected (as shown in Figure 15G,H). The DN‐900 shows excellent rate performance, i.e., the specific capacities of 370.8, 316.3, 266.3, 233.8, and 202.9 mAh g^−1^ at 0.1, 0.2, 0.5, 1, and 2A g^−1^, respectively, which is better than that of the DN‐800 and DN‐1000 (Figure 15I). In terms of cycle stability, even after up to 2000 cycles, the DN‐900 still shows a high reversible capacity of 193.5 mAh g^−1^ with a capacity retention close to 100%, significantly outperforming both DN‐800 and DN‐1000. The presence of large pores within the DGN‐900 structure facilitates electrolyte penetration and ion diffusion while serving as an electrolyte reservoir that accelerates rapid penetration or diffusion within the electrode. Additionally, the carbon nano walls between adjacent large pores effectively reduce the diffusion length for potassium ions to the nanometer scale, enabling enhanced ion transfer ability, particularly under high current density conditions. Moreover, the large pores also accommodate changes in electrode structure by providing sufficient pore volume that accommodates fluctuations during potassium ion (de)intercalation processes. This buffering effect helps relieve stress, maintain electrode structure stability, and improve overall cycle stability.

In summary, pores of varying sizes and structures exhibit distinct functions. The presence of a multilevel pore structure can result in a synergistic effect, providing abundant storage and diffusion pathways for potassium ions, thereby optimizing their storage and transport properties. The combination of macropores, mesopores, and micropores within the multilevel pore structure offers diverse diffusion routes and storage sites for potassium ions, enabling the battery to maintain excellent performance at different current densities. Under high current densities, large pores and mesoporous pores facilitate rapid transfer of potassium ions to meet the demands of fast charging and discharging. While at low current density, micropores and other pore structures can fully store potassium ions and increase the capacity.

Besides carbon materials, Liu explored the effect of pore size and pore structure in α‐Fe_2_O_3_ electrodes on the potassium storage.^[^ ^180^ ^]^ The material exhibits a pure phase with sea urchin‐like particles composed of acicular microcrystals, displaying a uniform structure. The electrode's pore structure varied with grinding time: S1 contained predominantly closed and semi pores, S2 exhibited diverse pore types, while S3 primarily consisted of large pores. It was observed that both the type and topological structure of the pores significantly influenced the potassium storage. An abundance of pore types contributed to enhancing initial coulomb efficiency and capacity, whereas a larger pore structure facilitated cycling stability under high current densities. Amongst these variations, S2 demonstrated the highest initial coulomb efficiency (83.1%) as well as the highest capacity (362.6mAh g^−1^) after 1000 cycles due to its plentiful range of pore types, which promoted stable SEI film formation. On the other hand, S3 displayed superior cycle performance at high rates (14A g^−1^).

In addition to pore size, different types of pores also influence the storage mechanism of K^+^ in the material. Zhang studied the storage mechanism of potassium ions for hard carbon anodes in the low‐voltage region.^[^ ^181^ ^]^ Hard carbon (HC‐1300) was prepared from pistachio shells through ball milling, sintering, and hydrochloric acid treatment, while lignin‐derived hard carbon (Lig‐HC) was prepared for comparative study. There are isolated graphene layers and small graphite domains in HC‐1300 samples, with a partially ordered structure. There are abundant micropores, mesopores, and macropores in HC‐1300, with a 158.1 m^2^g^−1^ high specific surface and a 0.043 cm^3^ g^−1^ total pore volume. These abundant pores provide an ideal platform for studying the potassium ion storage mechanism. It was observed that potassium ion storage primarily occurs through surface adsorption before reaching 0.5 V, followed by intercalation into graphite intercalation compounds (GICs) at ≈5 mV versus K^+^/K. After a constant‐voltage discharge (CVDi), it was detected a shift in K 2p peak to K metal by XPS, indicating the existence of quasi‐metallic K nanoclusters formed by filling nanopores with potassium ions. The mixed behavior of “intercalation and pore filling” was confirmed by electron paramagnetic resonance signals detected during cycling tests. Intercalation starts prior to reaching 5 mV discharge potential, while CVDi promotes complete insertion of potassium ions. Furthermore, open and closed pores exhibit distinct effects on the storage mechanism: although chemical activation via KOH treatment can improve kinetics by reducing voltage hysteresis, it does not promote pore filling due to its reduction in closed pore volume (HC‐KOH closed pore volume is reduced to 0.02 cm^3^g^−1^ which is more than 50% less than that of HC‐1300). It is presumed that only closed pores can accommodate potassium ions. In the future, it is necessary to prepare rich closed‐pore hard carbon to increase capacity.

#### 5.3.3 Amorphous Materials

To a certain extent, amorphous materials are able to be conceptualized as a limiting case of nanocrystalline systems. When the size of nanoparticles is continuously reduced to atomic dimensions, they can be considered as amorphous materials. Compared with traditional bulk phase and even nanocrystalline materials, the storage and transport mechanisms in amorphous materials exhibit distinct differences, particularly for relatively large potassium ions. In amorphous materials, larger vacancies facilitate the insertion of larger potassium ions, enabling some previously inactive or low‐capacity crystalline materials to achieve enhanced potassium storage capacity. Interestingly, in certain cases, the potassium storage of crystalline material is also accomplished through an amorphous state transformation to accommodate larger ions. Furthermore, in the amorphous state, interlayer spacing or potassium ion channels may be greater than those in crystals, which reduces volume change and reduce stress during cycling, promote potassium ion transport, improve electro–chemo–mechanical coupling properties as well as cycle stability.

**Figure 16:**
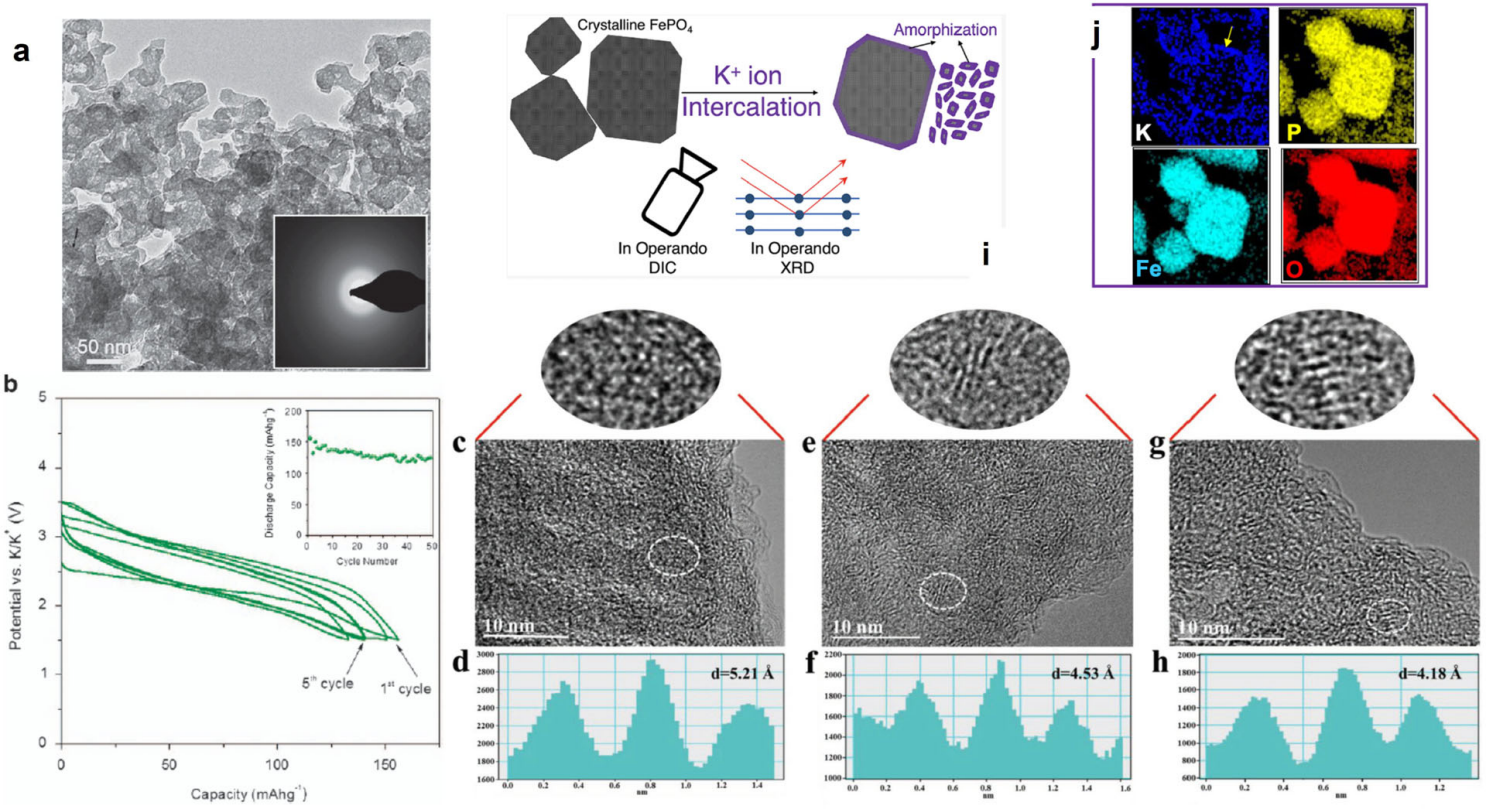
a) HRTEM image of amorphous FePO_4_. b) Potassium storage performance in amorphous FePO_4_. Reproduced with permission.^[^ ^56^ ^]^ Copyright 2014, Springer Nature. HRTEM images and the corresponding interlayer spacing of c,d) the original OMC, e,f) after full potassiation, and g,h) after full depotassiation. Reproduced with permission.^[^ ^62^ ^]^ Copyright 2018, Wiley‐VCH. i) Schematic illustration of in situ probing K^+^ intercalation‐induced amorphization in crystalline FePO_4_. j) Elemental analysis of Fe, P, K, and O in FePO_4_. Reproduced with permission.^[^ ^182^ ^]^ Copyright 2021, ACS.

The electrochemical energy storage through intercalation reactions in crystalline electrode materials is significantly linked to guest ions’ sizes. For example, the storage behaviors of olivine FePO_4_ in terms of Li^+^, Na^+^, and K^+^ are totally different. Kim et al. synthesized a porous amorphous FePO_4_ (**Figure** **16**a), which is a potential host for the intercalation of large K^+^. Its capacity is 156 mAhg^−1^ at 5 mAg^−1^, as demonstrated in Figure 16b.^[^ ^56^ ^]^ In another investigation, a crystalline FePO_4_ is prepared by electrochemically removing lithium from Olivine LiFePO_4_. Afterward, K^+^ was inserted into the FePO_4_ host. The strain and structure changes during the electrochemical process were in situ investigated by digital image correlation (DIC) and XRD (Figure 16i,j). A crystalline FePO_4_ transformed to an amorphous state in the first cycle, exhibiting no obvious changes with extended cycling. The strain analysis showed a distinct peak at specific potentials for intercalation of potassium, indicating that the amorphous phase FePO_4_ exhibits reversible redox chemistry.^[^ ^182^ ^]^

Crystalline and amorphous carbon materials show similar results. The crystalline carbon, commonly referred to as graphite, serves as a prevalent anode for K‐ion batteries. However, given the pronounced ionic size disparity between K^+^ and Li^+^, the intercalation process of potassium into graphite leads to considerable significant volumetric alterations, thereby leading to diminished capacity, inferior rate performance, and compromised cycle stability. Guo et al. prepared an amorphous ordered mesoporous carbon (OMC) as a potassium anode.^[^ ^62^ ^]^ For graphite, the interlayer space is 3.35 Å, where K ion can insert into the interlayer spacing to form KC_8_ with a capacity of 279 mAh g^−1^. After full potassiation, 59.7% enhancement of the graphite interlayer distance is observed, which is six times larger than that of lithiation (10.7%). Moreover, during the repeated charge and discharge process, the interlayer structure of graphite will collapse. However, for OMCs anodes, the situation is different and the electro–chemo–mechanical coupling properties have been much improved. For amorphous OMC, there is no crystallographic order in the long range, while the nominal interlayer distance is ≈5.21 Å in a short range (Figure 16c–h). After potassiation, the interlayer spacing is ≈4.53 Å, which is larger than the potassium ion’ size; while after depotassiation, the interlayer spacing decreases to 4.18 Å (≈7% variations). The interlayer spacing of amorphous OMC, compared with graphite, is much bigger, which possesses a higher capacity for accommodating K^+^ and can effectively adjust to changes in interlayer spacing during (de)intercalation of K^+^. The capacity of OMC is 257 mA h g^−1^ at 0.05 A g^−1^, and maintains 147 mA h g^−1^ at 1 A g^−1^ for more than 1000 cycles.

#### 5.3.4 Variations of Phase Transformation Mechanism

The phase transition mechanism of electrodes is closely linked to sizes, exemplified by the size effect observed in LiFePO_4_ (LFP), which has been emphasized in lithium‐ion batteries.^[^ ^44^, ^183^, ^184^, ^185^, ^186^, ^187^ ^]^ Typically, as particle size decreases, the ratio of the solid solution region to the two‐phase (or multiphase) phase transition region will change, leading to a gradual reduction in the two‐phase (multiphase) region and a corresponding expansion of the single‐phase solid solution region. In certain instances, the two‐phase region may entirely vanish, transitioning to a single‐phase solid solution mechanism. Distinct phase transition mechanisms are associated with varying storage mechanisms, transport dynamics and electro–chemo–mechanical coupling properties, particularly significant for potassium ions due to their larger ionic radii.^[^ ^188^ ^]^

**Figure 17:**
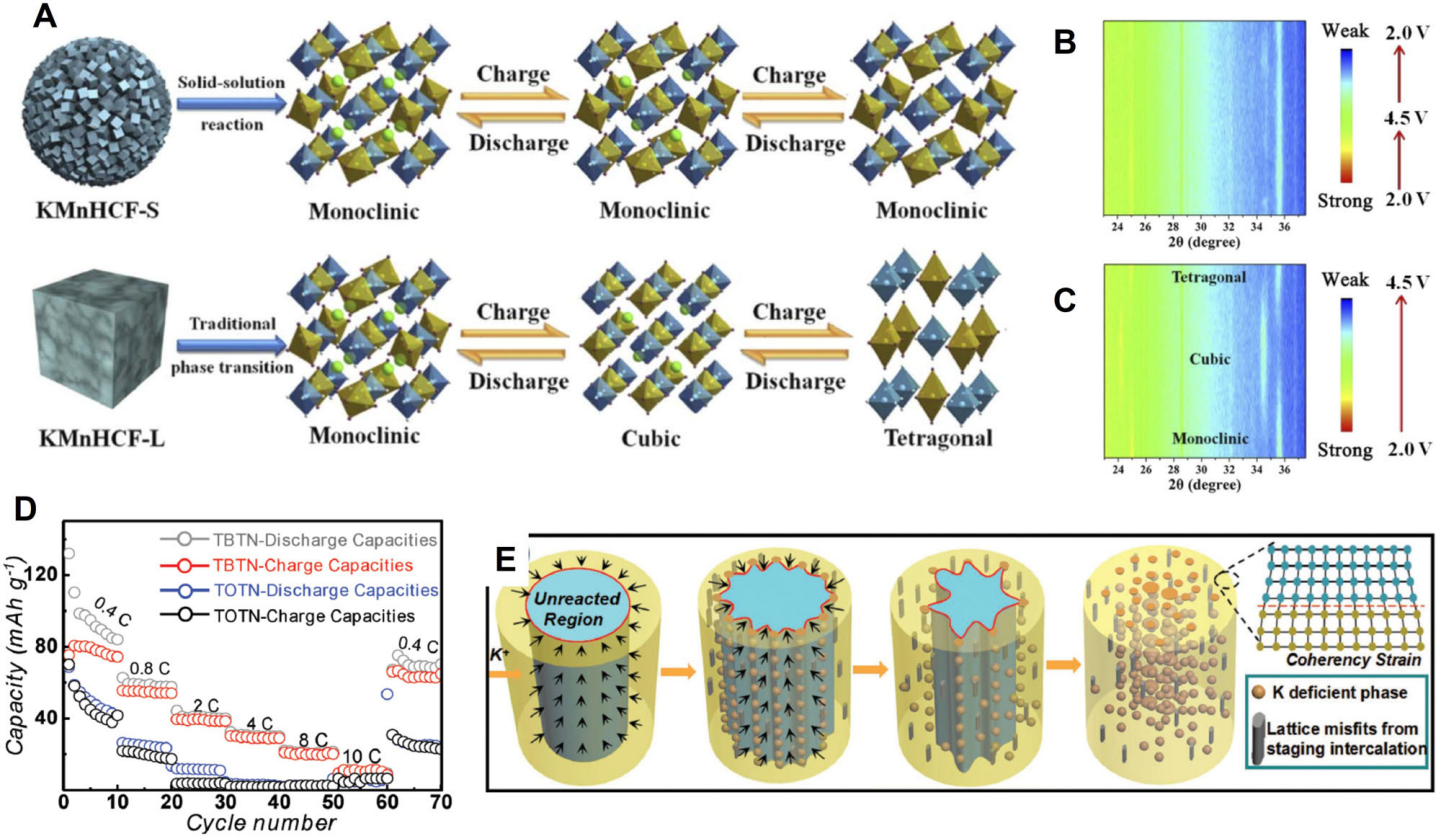
A) Schematic comparing solid‐solution reaction mechanisms with phase‐transition pathways. In situ XRD for B) KMnHCF‐S and C) KMnHCF‐L. Reproduced with permission.^[^ ^189^ ^]^ Copyright 2022, RSC. D) Rate capability of TOTN and TBTN. E) Schematic representations of intragranular particle generation. Reproduced with permission.^[^ ^190^ ^]^ Copyright 2018, Wiley‐VCH.

Prussian Blue Analogues (PBAs) are very suitable for the K storage owing to a 3D open framework. Potassium‐rich Manganese‐Prussian Blue (KMnHCF) exhibits a high operating voltage and significant capacity. Nevertheless, cyclic stability of the material is compromised due to the phase transition induced by the Jahn‐Teller effect. Wang et al. successfully synthesized hollow nanospheres (KMnHCF‐S) comprised of ultrasmall KMnHCF nanocube subunits.^[^ ^189^ ^]^ By reducing the particle size, it exhibits the solid solution reaction instead of phase separation, thereby mitigating the Jahn‐Teller effect and enhancing the electrochemical performance. The KMnHCF‐S structure consists of ultrasmall nanocube subunits ≈9 nm in diameter, featuring a hollow core and a rough exterior. In contrast, the KMnHCF‐M nanocube subunits measure ≈20 nm, while the KMnHCF‐L subunits range from 90 to 120 nm. In situ XRD analysis reveals that KMnHCF‐L and KMnHCF‐M undergo reversible phase transitions (monoclinic – cubic – tetragonal) during charging and discharging. Conversely, KMnHCF‐S demonstrates a solid solution reaction without evident phase change, maintaining nearly constant lattice parameters. This characteristic prevents the formation and migration of two‐phase interfaces, improves electro–chemo–mechanical coupling properties, reduces coherent strain, effectively suppressing the Jahn‐Teller effect, and leading to superior cyclic performance (**Figure** **17**A–C). At 0.05 A g^−1^, the capacity retention reaches 93.3% for 200 cycles. While at 0.1 A g^−1^, the capacity retention remains at 91.9% even after 1000 cycles. First‐principles calculations indicate that the bond lengths change significantly during the phase transition, whereas minimal changes occur in the single‐phase reaction. In the latter, the change of cell volume is small and the energy for potassium ion (de)intercalation is lower, which is helpful to improve the rate performance. At high current densities, such as 1, 2, and 3 A g^−1^, it maintained at 79, 62, 51 mAh g^−1^ in capacity. In addition, the solid solution reaction also avoids orbital splitting, thus suppressing the Jahn‐Teller distortion as well.

As previously discussed, reducing particle size to expand the solid solution domain represents a promising material design strategy. The presence of coherent strain significantly influences the small size effect, extending the solid solution range and inhibiting phase separation. Wang et al. synthesized K_2_Ti_6_O_13_ nanowires of varying diameters and examined the impact of size on the phase transition mechanism.^[^ ^190^ ^]^ They also explored the effect of stress on the solid solution behavior of potassium ion intercalation in K_2_Ti_6_O_13_ nanowires of different diameters, specifically the size‐dependent properties of coherent strain. Notably, K_2_Ti_6_O_13_ nanowires with an average diameter of ≈5.5 nm exhibit a high initial reversible depotassiated capacity, achieving a capacity of ≈120 mAh g^−1^ at 0.2C and high‐rate capability (Figure 17D). This enhanced capacity, rate performance, and improved cyclic stability can be attributed to the solid solution intercalation mechanism of potassium ions and the reduction of incoherent interfaces after potassiation, and improvement of electro–chemo–mechanical coupling properties. In contrast, 38 nm K_2_Ti_6_O_13_ nanowires display enrichment of intragranular particles in the potassic particles, suggesting strain‐accommodating misfit or dislocation during solid solution intercalation (Figure 17E). Coherent strain energy effectively enhances battery performance by maintaining a coherent interface; however, particle sizes exceeding a critical threshold result in irreversible capacity loss. Consequently, decreasing the size of the intercalated compound below this critical value to preserve coherence allows the coherent strain energy to facilitate reversible energy storage, enhancing both cycle and rate capability. Conversely, when the particle size surpasses the critical value, larger particles generate more incoherent interfaces during intercalation, leading to significant irreversible capacity degradation.

**Figure 18:**
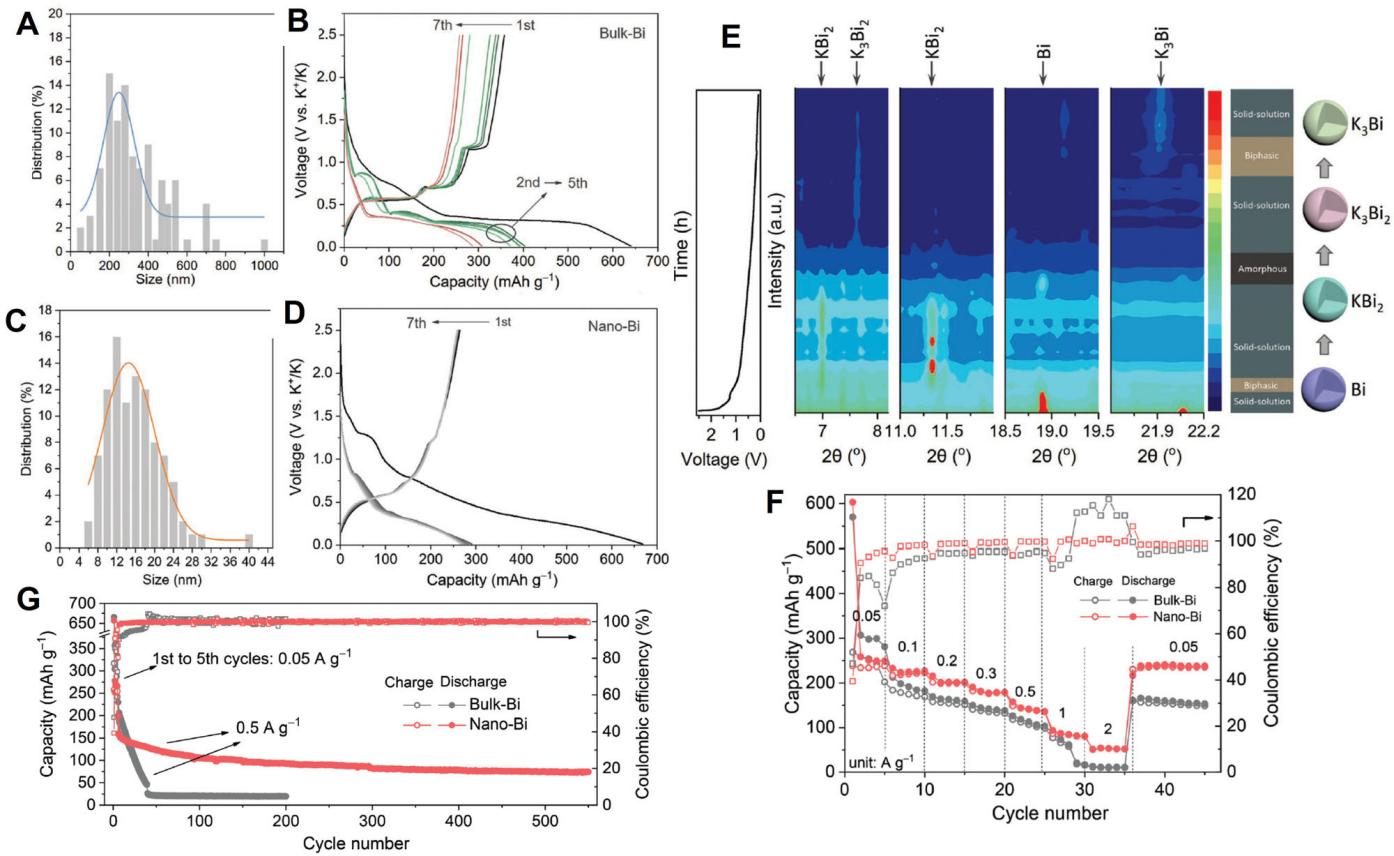
A) Bi particle size distribution with a mean value of ≈250 nm. B) Galvanostatic charge–discharge profiles for the first seven cycles (0.05 A g^−1^) for Bulk‐Bi. C) Bi particle size distribution with a mean value of ≈15 nm. D) Galvanostatic charge–discharge curves for the first seven cycles (0.05 A g^−1^) for Nano‐Bi. E) Coupled voltage profile and SXRD contour analysis of nano‐Bi during first potassiation. F) Rate performance for Bulk‐ and Nano‐Bi. G) Long cycling performance for Bulk‐ and Nano‐Bi. Reproduced with permission.^[^ ^191^ ^]^ Copyright 2022, Wiley‐VCH.

More importantly, by reducing the material size, the phase transition mechanism can be changed, thereby adjusting the dynamic rate‐determining step and effectively enhancing its potassium storage performance. Despite the high theoretical specific capacity of alloy‐type anode materials, significant lattice volume changes during potassium ion alloying result in the fracture of active particles and a decline in cycle life. The larger potassium ion radius exacerbates this issue. Reducing particle size is a widely adopted strategy to mitigate particle pulverization during cycling and to modulate the reaction mechanism. Kang et al. synthesized Bi anode materials of varying sizes, specifically 250 nm (bulk‐Bi) and 15 nm (nano‐Bi) (**Figure** **18**A,C).^[^ ^191^ ^]^ Ex situ synchrotron radiation XRD (SXRD) analysis of bulk‐Bi reveals that the initial discharge to 0.9 V is a pure Bi phase, and the discharge to 0.6 V shows the coexistence of three Bi‐K alloy phases, which is related to the different phase transition potentials of the alloy compounds, and there is also multiphase coexistence in deep discharge. The Bi phase is not completely potassiation at full discharge. The kinetic limitations of potassiation in larger particles lead to incomplete phase transitions and multiphase coexistence, resulting in abrupt voltage plateau changes (Figure 18B). In contrast, the initial potassiation of nano‐Bi, as revealed by in situ SXRD, follows a stepwise alloying mechanism with no detectable phase separation. Initially, a single‐phase reaction occurs, followed by a short two‐phase region. The subsequent intermediate product corresponds to the solid‐solution reaction in a wide region of single‐phase (Figure 18E). This differs from the multiphase coexistence observed during the potassiation of bulk‐Bi. HRTEM and XRD analyses of nano‐Bi under different charging states show that the dealloying reaction is reversible and a single‐phase solid solution. In contrast, the phase transition of bulk‐Bi is a two‐phase process, requiring new‐phase nucleation followed by intra‐particle growth. The different phase transition mechanisms can be attributed to distinct kinetic behaviors. Ion diffusion in bulk‐Bi is primarily governed by interface‐controlled reactions (ICR), whereas ion diffusion in nano‐Bi is predominantly driven by diffusion‐controlled reactions (DCR). Consequently, these differences result in varying diffusion anisotropies for each material and elucidate the fundamental reason behind the superior mechanical stability of nanoscale electrodes. EIS and GITT measurements show that nano‐Bi has faster electron and ion transport and better reaction kinetics. Nano‐Bi demonstrates superior rate performance compared to bulk‐Bi. As illustrated in Figure 18F, at 0.05, 0.1, 0.2, 0.3, 0.5, 1, and 2 A g^−1^, nano‐Bi exhibits capacities of 250, 225, 202, 181, 141, 84, and 52 mAh g^−1^, respectively. In contrast, bulk‐Bi shows capacities of 243, 178, 154, 135, 102, 66, and 11 mAh g^−1^ under the same conditions. Furthermore, nano‐Bi exhibits outstanding cycling stability, maintaining 74 mAh g^−1^ after 550 cycles, whereas bulk‐Bi experiences a significant capacity fade and battery failure after just 40 cycles (Figure 18G).

## 6 Conclusion and Outlook

**Figure 19:**
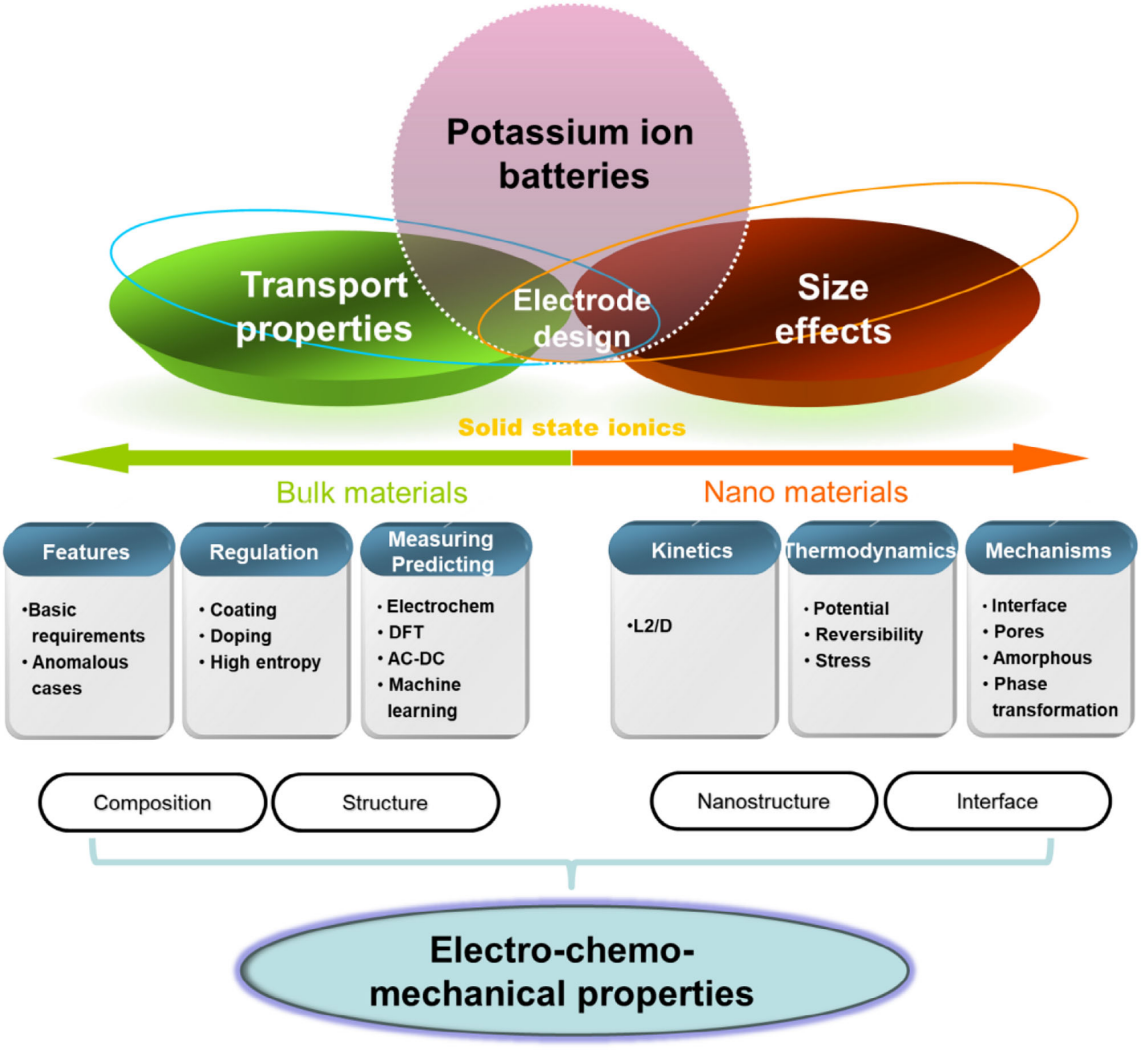
The significance of transport properties, size effects, and electro–chemo–mechanical properties of potassium electrodes, along with a schematic diagram illustrating the key contents reviewed in this paper.

In this review, we first compare the similarities and differences in the chemical and physical properties of potassium relative to other alkali metals such as lithium and sodium. We then gave a comprehensive evaluation of the advantages and disadvantages of K‐ion batteries and potassium‐based batteries, assessing their future development from five dimensions: energy density, power density, cycle life, safety, and cost. A critical challenge for potassium‐ion battery electrode materials is the poor transport kinetics of potassium ions within these materials and the structural damage caused by significant volume changes during (de)intercalation. Therefore, addressing this issue requires clarifying and improving transport properties, size effects, and electro–chemo–mechanical properties. This review systematically examines key aspects of potassium electrode materials from a unique perspective, as summarized in **Figure** **19**.

From a transport properties perspective, potassium ions, due to their larger size, generally exhibit inferior transport properties compared to lithium and sodium ions. For some materials, there is no electrochemical activity. Therefore, when selecting or developing new potassium electrode materials, preference should be given to materials with larger ion transport channels or wider interlayer spacing. Additionally, factors such as appropriate framework dimensions, ion‐framework interactions, and the synergy between thermodynamics and kinetics must be comprehensively considered. For controlling transport characteristics, coating and doping are effective intrinsic methods. The application of high‐entropy electrode design strategies in potassium‐ion systems also deserves special attention. Electrochemical methods are commonly used for measuring transport properties; however, optimizing the measurement of electrode physical parameters is necessary to minimize errors and inconsistencies. DFT can predict transport properties of electrodes, especially for novel materials, by describing and determining their transport pathways at the atomic level. The combined AC‐DC method can accurately measure ionic and electronic conductivities separately, aiding in establishing defect chemistry models, although research in this area remains less developed compared to lithium and sodium systems. Machine learning can integrate these methods, combining theoretical and experimental approaches to accelerate material development and performance prediction, making it particularly noteworthy for the emerging K‐ion battery field.

The transport of potassium ions exhibits distinct characteristics at various levels, and future research could investigate this phenomenon from multiple perspectives. 1) Bulk level (macroscopic level). The bulk‐phase migration of K ions reflects their fundamental transport mechanism. The storage mechanism significantly influences the K^+^ transport behavior in the bulk phase. One of the simplest and most easily described cases is the single‐phase storage, that is, the solid solution mechanism, which only needs to consider the bulk phase diffusion of potassium ions. However, in many cases, a combination of single‐phase and two‐phase transformation mechanisms exists, with their proportions closely linked to size effects. In such instances, both single‐phase diffusion and phase transition situations need to be considered; specifically, one must examine the diffusion characteristics within each phase as well as specific phase transition mechanisms, like how two‐phase interfaces impact transport phenomena. Apart from two‐phase transitions, multiphase mechanisms are also common for storing potassium ion electrodes (e.g., conversion reaction in potassium electrodes). Due to involvement with multiphase nucleation and diffusion processes, its transport characteristics will be more complex. 2) Bulk phase versus interface. In many cases, the coexistence of bulk phase and interface transport is observed, particularly as the size of electrode materials gradually decreases, resulting in a higher proportion of interfaces. Therefore, it becomes crucial to pay special attention to the ion storage and transport at the interface. As mentioned earlier, both the transport characteristics and size effects of materials influence the proportion of storage mechanisms between bulk phase and interface. Consequently, these differences in storage mechanisms subsequently impact the transport characteristics. In addition, for interface transport, by constructing different interfaces or interface heterojunctions, it is possible to obtain faster transport dynamics than the bulk phase. 3) Material level versus electrode sheet level. In addition to considering the transport characteristics of the material itself, it is also crucial to pay attention to the transport at the electrode sheet level, as it is closer to the actual situation. When examining the electrode sheet, one must not only consider the K^+^ transport within the electrodes, such as the issues of phase transformation, the influence of the bulk phase and the interface, but also take into account its transport behaviors through conductive agents, binders, and various interfaces. This situation adds further complexity.

From the perspective of size effects, reducing material sizes serves as an effective external control method for enhancing transport properties. This approach can significantly improve the transport kinetics of materials, particularly for larger potassium ion transport. Additionally, size reduction can modulate the thermodynamic and intrinsic characteristics of materials, even potentially enabling materials with no electrochemical activity to exhibit superior performance. This approach can be used as an effective screening tool for identifying new potassium electrode materials. For instance, reducing the size varies the equilibrium potential of the material, while the inherent size of potassium ions also influences this potential; thermodynamic reversibility can be enhanced, leading to the possibility of achieving a fully reversible potassium electrode; the intrinsic stress characteristics of potassium electrodes are also altered. Moreover, size effects can induce significant changes in potassium ion storage and transport mechanisms: interface storage and transport effects are amplified; the distinct roles of hierarchical pores (macropores, mesopores, micropores, and composite pores) become more pronounced; amorphous materials gain unique advantages in potassium ion storage; phase transition mechanisms change, as well as rate‐determining step in kinetics.

For the size effects of potassium electrodes, from a fundamental research perspective, it is essential to investigate the unique thermodynamic, kinetic, and storage/transport mechanism changes in materials at extremely small dimensions. This exploration aims to uncover ultimate limits and performance boundaries in potassium electrodes, thereby guiding material design and new material screening. From an application standpoint, it is crucial to mitigate challenges such as preparation difficulties, costs, and side reactions associated with small sizes. Rational design should be conducted based on the intrinsic transport characteristics of the material to achieve optimal nanoscale efficiency. Additionally, various micro and nano regulation techniques should be employed to maximize the advantages of both micron and nano‐scale materials, leading to the development of micro–nano composite electrode materials and electrode sheet structures that provide viable solutions for the industrialization of K‐ion batteries.

The electro–chemo–mechanical properties of potassium electrodes represent a critical challenge that significantly limits their performance. Combined with the understanding and improvement of transport properties and size effects, the electro–chemo–mechanical coupling behaviors can also be effectively improved. The electro–chemo–mechanical coupling behaviors of potassium electrodes can be improved and optimized through strategies such as composition–structure regulation (e.g., doping, high‐entropy electrode design), nanostructure engineering (e.g., nanoscale design, porous structures, amorphous phases, phase transition mechanism regulation), and interface engineering (e.g., CEI/SEI composition optimization and mechanical strength enhancement). In the future, it is important to further investigate high‐stability potassium electrode materials and develop novel electrode materials with an emphasis on adaptive volume adjustment capabilities. Simultaneously, advanced characterization techniques and multi‐scale simulation approaches should be further refined, such as in situ mechanical testing combined with multi‐scale simulations, to elucidate the electro–chemo–mechanical coupling behaviors under real‐time conditions.

## Conflict of Interest

The authors declare no conflict of interest.
